# Maximal Dissipation and Well-Posedness of the Euler System of Gas Dynamics

**DOI:** 10.1007/s00205-026-02205-4

**Published:** 2026-06-03

**Authors:** Eduard Feireisl, Ansgar Jüngel, Mária Lukáčová-MedviĎová

**Affiliations:** 1 Institute of Mathematics of the Academy of Sciences of the Czech Republic, Žitná 25, 115 67 Praha 1, Czech Republic; 2 Institute of Analysis and Scientific Computing, TU Wien, Wiedner Hauptstr. 8–10, 1040 Wien, Austria; 3 Institute of Mathematics, Johannes Gutenberg-University Mainz, Staudingerweg 9, 55 128 Mainz, Germany; 4 Erwin Schrödinger International Institute for Mathematics and Physics, Boltzmanngasse 9, 1090 Wien, Austria

## Abstract

We show that any dissipative (measure–valued) solution of the compressible Euler system that complies with Dafermos’ criterion of maximal dissipation is necessarily an admissible weak solution. In addition, we propose a simple, at most two-step, selection procedure to identify a unique semigroup solution in the class of dissipative solutions to the Euler system. Finally, we introduce a refined version of Dafermos’ criterion yielding a unique solution of the problem for any finite energy initial data.

## Introduction

Mathematical models of perfect fluids in continuum mechanics exhibit the well known difficulties including formation of singularities and ill posedness with respect to the data. An iconic example is the *barotropic Euler system* of gas dynamics describing the time evolution of the density $\varrho = \varrho (t,x)$ and the velocity $\textbf{u}= \textbf{u}(t,x)$ of a compressible perfect (inviscid) fluid:

1.1 $$\begin{aligned} \partial _t \varrho + \textrm{div}_x(\varrho \textbf{u})&= 0, \end{aligned}$$

1.2 $$\begin{aligned} \partial _t (\varrho \textbf{u}) + \textrm{div}_x(\varrho \textbf{u}\otimes \textbf{u}) + \nabla _xp(\varrho )&=0. \end{aligned}$$

Here *p* is the pressure depending solely on the fluid density, whereas the thermal effects are ignored. The fluid occupies a domain $\Omega \subset R^d$, d=1,2,3, and the evolution is considered in the time interval $t \in [0, \infty )$. Relevant initial/boundary data must be prescribed to obtain, at least formally, a mathematically well-posed problem. Here, we consider the initial conditions

1.3 $$\begin{aligned} \varrho (0, \cdot ) = \varrho _0,\ (\varrho \textbf{u})(0, \cdot ) = \textbf{m}_0, \end{aligned}$$

together with the impermeability boundary condition

1.4 $$\begin{aligned} \textbf{u}\cdot \textbf{n}|_{\partial \Omega } = 0. \end{aligned}$$

More general boundary conditions are discussed in the monograph [4, Chapter 3,4].

We recall the well known facts concerning solvability of the Euler system:

- Problem (1.1)–(1.4) admits a local in time classical solution provided the initial data enjoy Sobolev regularity $W^{k,2}$, $k > 1 + \frac{d}{2}$, the initial density $\varrho _0$ is bounded below away from zero, and the initial velocity (momentum) $\textbf{m}_0$ satisfies the relevant compatibility condition, see, e.g., [4] or [34] for a proper interpretation of the compatibility conditions.
- Smooth solutions develop shock type singularities in a finite time for a vast class of initial data, see e.g. Dafermos [15], Smoller [35].
- Singularities in the form of implosions may develop in the physically relevant 3D geometry, see Buckmaster et al. [9], Cao-Labora et al. [10], Merle et al. [32, 33].

### Weak Solutions

To incorporate possible physically relevant singularities, the concept of *weak* distributional solutions was introduced, along with an admissibility condition

1.5 $$\begin{aligned} \partial _t E(\varrho , \textbf{u}) + \textrm{div}_x\left[ \Big ( E(\varrho , \textbf{u}) + p(\varrho ) \Big ) \textbf{u}\right] \le 0, \end{aligned}$$

where

$$\begin{aligned} E(\varrho , \textbf{u}) = \frac{1}{2} \varrho |\textbf{u}|^2 + P(\varrho ),\ P'(\varrho ) \varrho - P(\varrho ) = p(\varrho ) \end{aligned}$$

represents the energy of the system. The weak formulation of (1.1), (1.2), along with the admissibility condition (energy inequality) (1.5), is capable to identify a unique solution in many physically relevant situations, in particular the so–called Riemann problem in the 1-D geometry, see e.g. Bianchini, Bressan [5] or the monograph by Dafermos [15].

The same Riemann problem considered in the multi-D setting, however, gives rise to a large variety of different but still admissible weak solutions “constructed” by the method of convex integration, see e.g. Chiodaroli, De Lellis, and Kreml [12] among many others. Apart from the Riemann problem the ill-posedness of the barotropic Euler system was observed in the seminal paper De Lellis and Székehylidi [16]. More recently, ill posedness was shown even in the class of Hölder continuous admissible solutions, see Giri, Kwon [25].

Despite the abundance of weak solutions for certain “wild” data, a simple question of *existence* of an admissible weak solution for *any* finite energy initial data remains open, even in the incompressible case, cf. Wiedemann, Székelyhidi [36], and Wiedemann [37]. To fill this gap, in particular when identifying the limits of numerical approximations, a larger class of dissipative (measure–valued) solutions is needed, see e.g. [23].

### Dissipative Solutions

The origin of dissipative solutions goes back to DiPerna [18], see also DiPerna, Majda [17], and their concept of measure–valued solution to capture the qualitative properties of oscillatory approximations. Unfortunately, the class of Young measures and their various extensions to include possible concentrations (see e.g. Alibert, Bouchitté [2]) seems to be too large to give rise to a unique solution. In particular, the unphysical “numerical” oscillations may create different measure valued solutions depending on the approximation method. Instead, only certain physically observable quantities should be invariant and recovered independently of a specific approximation process. In [8], only the expected value (with respect to the associated Young measure) of the density ϱ, the momentum $\textbf{m}= \varrho \textbf{u}$, and the scalar quantity $\mathcal {E}$ representing the total energy were proposed as a new concept of *dissipative solution* of the Euler system.

Roughly speaking, dissipative solutions solve in the distributional sense an extended Euler system

1.6 $$\begin{aligned} \partial _t \varrho + \textrm{div}_x\textbf{m}&= 0, \end{aligned}$$

1.7 $$\begin{aligned} \partial _t \textbf{m}+ \textrm{div}_x\left( \frac{\textbf{m}\otimes \textbf{m}}{\varrho } \right) + \nabla _xp(\varrho )&= - \textrm{div}_x\mathcal {R}, \end{aligned}$$

with a tensor $\mathcal {R} \ge 0$ called *Reynolds stress*. In addition, there is a new variable $\mathcal {E}$ called *total energy* – a scalar quantity satisfying

1.8 $$\begin{aligned} \frac{\textrm{d}}{\,\textrm{d} t } \mathcal {E} \le 0. \end{aligned}$$

Moreover, the energy defect

$$\begin{aligned} \mathcal {E} - \int _{\Omega } E(\varrho , \textbf{m}) \ \,\textrm{d} {x} \ge 0 \end{aligned}$$

controls the amplitude of the Reynolds stress, more specifically,

1.9 $$\begin{aligned} \mathcal {E} - \int _{\Omega } E(\varrho , \textbf{m}) \ \,\textrm{d} {x} \ge c(p) \int _{\overline{\Omega }} \textrm{d}\ \textrm{trace}[\mathcal {R}] , \end{aligned}$$

where the constant c(p)>0 is determined in terms of the structural properties of the equation of state p=p(ϱ). The integral on the right–hand side of (1.9) is computed in terms of the measure $\textrm{trace}[\mathcal {R}]$. We call the quantity $\int _{\Omega } E(\varrho , \textbf{m}) \ \,\textrm{d} {x}$ *mean energy*. Admissible weak solutions (with a non–increasing total energy) are therefore characterized by the equality

$$\begin{aligned} \mathcal {E}(t) = \int _{\Omega } E(\varrho , \textbf{m})(t, \cdot ) \ \,\textrm{d} {x} \ \text{ valid } \text{ for } \text{ a.a. }\ t \ge 0. \end{aligned}$$

We refer to Sect. 2.1 for a precise definition of dissipative solutions.

#### Remark 1.1

The terminology we use is reminiscent of the turbulence modelling. Note, however, that the meaning of the quantities like Reynolds stress or energy defect are rather different in the context of the (inviscid) Euler system and the models of viscous fluids used to describe turbulence.

#### Comparison with Other Concepts of Generalized Solutions

Recently, Eiter and Lasarzik [19] proposed a general approach applicable to systems of conservation laws. In the particular case of the barotropic Euler system, their concept of weak solution coincides with the dissipative solutions introduced in the previous section, see [19, Theorem 5.8].

Inspired by the definition of variational solutions to the incompressible Euler system by Lions [30], Lasarzik [29] introduced a similar concept of solutions based on the mere satisfaction of the relative energy inequality. A more sophisticated approach of similar “variational” character was proposed also by Brenier [6]. The resulting problem is quite elegant and its solution boils down to resolving a kind of convex variational problem. As the relative energy inequality is available also for the measure valued/dissipative solutions of the Euler system, cf. e.g. Gwiazda, Świerczewska-Gwiazda, and Wiedemann [26] or [22], a similar approach can also be developed also for the present problem. The class of possible solutions, however, is quite large and the desired solution of the Euler system is correctly identified only if it is smooth globally in time. As pointed out by Brenier [6], the resulting solutions may fail in attaining the prescribed initial data, and, in general, they may not be even (weakly) continuous with respect to time. In particular, the solution may not coincide with the smooth one on its life–span, see the example of Burgers equation discussed in [6].

#### Basic Properties of Dissipative Solutions

The dissipative solutions enjoy the following properties:

- **Global existence.** Given the initial data
  $$\begin{aligned} \varrho _0, \ \textbf{m}_0, \ \text{ and } \ \mathcal {E}_0 \ge \int _{\Omega } E(\varrho _0, \textbf{m}_0) \ \,\textrm{d} {x}, \end{aligned}$$
  there exists a global–in–time dissipative solution of the Euler system. The functions $\varrho (t, \cdot )$, $\textbf{m}(t, \cdot )$ are weakly continuous in time and satisfy the initial conditions in the weak sense. Moreover, the total energy $\mathcal {E}$ can be identified with a non–increasing càglàd function of the time, with $\mathcal {E}(0) \equiv \mathcal {E}(0-) = \mathcal {E}_0$, see [8].
- **Compatibility.** If
  1.10 $$\begin{aligned} \mathcal {E}_0 = \int _{\Omega } E(\varrho _0, \textbf{m}_0) \ \,\textrm{d} {x} \end{aligned}$$
  and ϱ, $\textbf{m}$ are continuously differentiable on the time interval [0, *T*), ϱ>0, then $\mathcal {R} = 0$ and ϱ, $\textbf{m}$ represent a classical solution on [0, *T*), see e.g. [22].
- **Weak–strong uniqueness.** If (1.10) holds, and the Euler system admits a continuously differentiable (classical) solution in [0, *T*), then $\mathcal {R} = 0$ and any dissipative solution coincides with the classical solution in [0, *T*).
- **Asymptotic regularity.** We define a partial ordering of the class of dissipative solutions emanating from the same initial data,
  1.11 $$\begin{aligned} (\varrho ^1, \textbf{m}^1, \mathcal {E}^1) \prec (\varrho ^2, \textbf{m}^2, \mathcal {E}^2) \ \Leftrightarrow \ \mathcal {E}^1 (t) \le \mathcal {E}^2(t) \ \text{ for } \text{ all }\ t > 0. \end{aligned}$$
  We say that a dissipative solution is *admissible* if it is *minimal* with respect to ≺. Admissible dissipative solutions exist for any finite energy initial data, see [8]. As shown in [20], the energy defect of admissible dissipative solutions vanishes in the long run, more specifically,
  1.12 $$\begin{aligned} \mathcal {E}(t) - \int _{\Omega } E(\varrho , \textbf{m}) (t, \cdot ) \ \,\textrm{d} {x} \rightarrow 0 \ \Rightarrow \ \int _{\overline{\Omega }} \textrm{d}\ \left| \mathcal {R}(t, \cdot ) \right| \rightarrow 0 \ \text{ as }\ t \rightarrow \infty . \end{aligned}$$

Fluids arising in real world applications are always viscous—they dissipate mechanical energy in some sense. The Euler system should be therefore understood as a zero viscosity limit. The same is true for numerical approximations containing “artificial” numerical viscosity as a stabilizing mechanism. On the one hand, a vanishing viscosity limit is a weak solution of the compressible Euler system only if the approximate solutions converge strongly (pointwise a.a.), see [21] or [22, Chapter 7, Theorem 7.5]. In particular, the weak convergence of approximate solutions observed for certain numerical schemes gives rise to truly dissipative solutions, meaning $\mathcal {R} \ne 0$, see Fjordholm et al. [24], Lukáčová et al. [31]. On the other hand, as we show in the present paper, any dissipative solution satisfying Dafermos’ admissibility criterion of maximal dissipation is necessarily a weak solution. The incompatibility of weak convergence and the principle of maximal dissipation may be seen as a paradigm shift in our understanding of what is a correct limit of numerical approximations of models involving perfect fluids, cf. also Chiodaroli and Kreml [13].

### Maximal Dissipation Principle, DiPerna’s Conjecture, Dafermos’ Admissibility Criterion

The first group of results obtained in the present paper concerns regularity of dissipative solutions.

It may seem that the dissipative solutions may deviate largely from the weak solutions due to a possibly large amplitude of the Reynolds stress $\mathcal {R}$. However, as we show in Sect. 3.1, there exist dissipative solutions with arbitrarily small amplitude of $\mathcal {R}$. Specifically, for any initial data and any δ>0, there exists a dissipative solution satisfying

$$\begin{aligned} \int _{\overline{\Omega }} \textrm{d}\ |\mathcal {R}(t, \cdot ) | \le \delta \ \text{ for } \text{ any }\ t \ge 0. \end{aligned}$$

The proof is based on suitable partial ordering of the set of dissipative solutions and an application of Zorn’s lemma.

Next, we recall DiPerna’s conjecture [18]:

Any measure–valued solution minimal with respect to ≺ is in fact a weak solution of the Euler system.

The validity of the above statement for the Euler system is still open. Motivated by the seminal work of Dafermos [14], we refine the relation ≺ introduced in (1.11) to

$$\begin{aligned} (\varrho ^1, \textbf{m}^1, \mathcal {E}^1)&\prec _\textrm{loc} (\varrho ^2, \textbf{m}^2, \mathcal {E}^2) \\&\Leftrightarrow \\ (\varrho ^1, \textbf{m}^1, \mathcal {E}^1)(t)&= (\varrho ^2, \textbf{m}^2, \mathcal {E}^2)(t) \ \text{ for }\ 0 \le t \le T, \ \mathcal {E}^1(t)< \mathcal {E}^2(t),\ T< t < T+ \delta , \end{aligned}$$

for some $0 \le T < \infty$, δ>0. In other words, the solutions $(\varrho ^1, \textbf{m}^1, \mathcal {E}^1)$, $(\varrho ^2, \textbf{m}^2, \mathcal {E}^2)$ coincide up to the time *T*, while $(\varrho ^1, \textbf{m}^1, \mathcal {E}^1)$ dissipates more energy than $(\varrho ^2, \textbf{m}^2, \mathcal {E}^2)$ on the interval (T,T+δ). Note carefully that the relation $\prec _\textrm{loc}$ is a strict partial order, meaning it is irreflexive, asymmetric, and transitive.

We say that a dissipative solution is *maximal dissipative* if it is minimal with respect to the ordering $\prec _\textrm{loc}$. Accordingly, maximal dissipative solutions comply with Dafermos’ admissibility criterion based on maximal dissipation. Our main result stated in Theorem 3.3 below asserts that any maximal dissipative solution is necessarily a weak solution of the Euler system with a non–increasing total energy. This can be seen as a rigorous verification of DiPerna’s conjecture (in the framework of Dafermos’ admissibility criterion) in the specific case of the barotropic Euler system.

### Selection Criteria

The second main topic discussed in the present paper is the choice of a proper selection criterion applicable to the class of dissipative solutions. The first step in this direction was undertaken in [8] (cf. also [7]), where the approach of Cardona and Kapitanski [11] motivated by the original work by Krylov [28] was adapted to the Euler system. The selection procedure consists in a *successive* minimization of a family of cost functionals of the type

$$\begin{aligned} \mathcal {F}_{n,m} = \int _0^\infty \exp (-\lambda _n t) F_m(\varrho , {\textbf {m}}, \mathcal {E}) \,\text {d} t , \quad \lambda _n > 0, \end{aligned}$$

where the family of bounded continuous functionals $F_m$ separates points in a suitable phase space. As a result, a *unique* semigroup selection is identified as the asymptotic limit of the process. The selected solutions depend in the Borel measurable way on the initial data.

The method is a bit awkward and suffers the obvious difficulties:

- Although the first functional may be clearly specified as
  1.13 $$\begin{aligned} \mathcal {F}_{1,1} = \int _0^\infty \exp (-t) \beta (\mathcal {E}(t)) \,\textrm{d} t , \end{aligned}$$
  where β is a bounded increasing function, the subsequent choice of $F_m$ as well as the exponents $\lambda _n$ is entirely arbitrary. Obviously, different choices may give rise to different limits and there is no clear indication of possible preferences.
- The infinite selection process is unlikely to be efficiently numerically implemented/tested.

We propose a different much simpler selection process consisting of only two steps. Similarly to [8], the first step is minimizing

$$\begin{aligned} \mathcal {F}_{1} = \int _0^\infty \exp (-t) \mathcal {E}(t) \,\textrm{d} t . \end{aligned}$$

In comparison with (1.13), the function β is missing, in particular, the functional is not bounded. In order to select a measurable minimizer, a slight change of the solution space with respect to [8] is needed. Note that $\mathcal {F}_1$ can be obviously written in the form

$$\begin{aligned} \mathcal {F}_{1} {=} \int _0^\infty \exp (-t) \Big [ \mathcal {E}(t) {-} \int _{\Omega } E(\varrho , \textbf{m})(t, \cdot ) \ \,\textrm{d} {x} \Big ] \,\textrm{d} t {+} \int _0^\infty \exp (-t) E(\varrho , \textbf{m}) (t, \cdot ) \,\textrm{d} t \end{aligned}$$

- the sum of the (weighted) time integral of the energy defect and the mean energy. There is a number of arguments discussed already in [8, Section 5.1] why $\mathcal {F}_1$ should be the first to minimize. In particular, the selected solutions are necessarily admissible (minimal) with respect to the relation ≺ introduced in (1.11).

We conclude the selection process by the second step, namely, minimizing

$$\begin{aligned} \mathcal {F}_2 = \int _0^\infty \exp (-t) \int _{\Omega } F(\varrho , \textbf{m}, \mathcal {E})(t) \ \,\textrm{d} {x} \,\textrm{d} t , \end{aligned}$$

where

$$\begin{aligned} F: R \times R^d \times R \rightarrow [0, \infty ) \end{aligned}$$

is a suitable strictly convex function. We may call *F* “entropy” associated to the problem. In contrast to [8], the functional

$$\begin{aligned} (\varrho , \textbf{m}, \mathcal {E}) \mapsto \int _{\Omega } F(\varrho , \textbf{m}, \mathcal {E})(t) \ \,\textrm{d} {x} \end{aligned}$$

is neither bounded nor continuous with respect to the weak topologies pertinent to the solution space. Accordingly, the main new issue to be properly addressed is the Borel measurability of the selected minimizer with respect to the data. While measurability of the solution sets in [8] was reduced to measurability of the solution mapping in the Hausdorff topology on compact subsets of the phase space, we have to use the Wijsman topology on convex closed sets of suitable $L^p$-spaces. Fortunately, under certain conditions, convergence in the latter is equivalent to the so–called Mosco convergence of closed convex sets suitable to establish continuity of the minimizers.

Once measurability is established, step 2 yields a minimizer of $\mathcal {F}_2$. As the cost functional is strictly convex, this concludes the selection process. Here, convexity of the set of all dissipative solutions emanating from the same initial data plays a crucial role. In sharp contrast with [8], ambiguity of the result is only due to the choice of the entropy *F*—a topic subject to future discussion.

As shown in Theorem 4.2, the above selection process gives rise to a unique solution in the class of admissible dissipative solutions that enjoys the semigroup property. We call this solution a *semigroup solution*.

### Maximal Dissipation Principle Revisited

Motivated by the selection procedure we introduce a concept of *absolute energy minimizer*

$$\begin{aligned} (\underline{\varrho }, \underline{\textbf{m}}, \underline{\mathcal {E}}) \end{aligned}$$

emanating from the initial data $(\varrho _0, \textbf{m}_0, \mathcal {E}_0)$, and satisfying

$$\begin{aligned} \int _0^\infty \exp (-\lambda t) \underline{\mathcal {E}}(t) \,\text {d} t \le \int _0^\infty \exp (-\lambda t) \mathcal {E}(t) \,\text {d} t \quad \text {for } \text { all }\ \lambda \ge \underline{\lambda }\end{aligned}$$

for any other solution $(\varrho , \textbf{m}, \mathcal {E})$ starting from the same initial data, where $\underline{\lambda } = \underline{\lambda } (\varrho , \textbf{m}, \mathcal {E})$ depends on the solution $(\varrho , \textbf{m}, \mathcal {E})$.

We show that for each initial data there exists at most one absolute energy minimizer. Moreover, any absolute energy minimizer is minimal with respect to the order $\prec _\textrm{loc}$; whence a weak solution of the Euler system with a non–increasing energy profile. Finally, we observe that any *global* minimizer with respect to the order $\prec _\textrm{loc}$ must be an absolute energy minimizer. This simple but quite important fact provides a local admissibility criterion applicable on any compact time interval [0, *T*] *independent* of the behaviour of solutions in the long run.

The rest of this paper is organized as follow: in Sect. 2, we introduce the class of dissipative solutions to the barotropic Euler system and review their basic properties. In Sect. 3, we state and prove our main results concerning the existence of dissipative solutions with arbitrarily small Reynolds stress (Theorem 3.1 and Corollary 3.2). Moreover, we show that any maximal dissipative solution is necessarily a weak solution (Theorem 3.3). The selection process to identify a unique semigroup solution is discussed in Sect. 4. In Sect. 5, we introduce the class of absolute energy minimizers and discuss their basic properties.

## Dissipative Solutions to the Barotropic Euler System

In this section, we introduce the class of dissipative solutions to the Euler system. For definiteness, we restrict ourselves to the isentropic state equation

2.1 $$\begin{aligned} p(\varrho ) = a \varrho ^\gamma ,\quad a> 0,\ \gamma > 1. \end{aligned}$$

Accordingly, the corresponding pressure potential is given by

$$\begin{aligned} P(\varrho ) = \frac{a}{\gamma - 1}\varrho ^\gamma ,\quad P'(\varrho ) \varrho - P(\varrho ) = p(\varrho ). \end{aligned}$$

The energy function is defined as

2.2 $$\begin{aligned} E(\varrho , {\textbf {m}}) = \left\{ \begin{array}{ll} \frac{1}{2} \frac{|{\textbf {m}}|^2}{\varrho } + P(\varrho ) & \ \text { if }\ \varrho > 0,\\ 0 & \ \text { if }\ \varrho = 0,\ {\textbf {m}}= 0, \\ \infty & \ \text { otherwise } \end{array} \right. ,\end{aligned}$$

Note that $E: R^{d + 1} \rightarrow [0, \infty ]$ is a convex l.s.c. function, strictly convex on its domain. Finally, we suppose that $\Omega \subset R^d$ is a bounded Lipschitz domain.

### Definition of Dissipative Solutions

The initial data $(\varrho _0, \textbf{m}_0, \mathcal {E}_0)$ belong to the class

2.3 $$\begin{aligned} \mathcal {D} = \left\{ (\varrho _0, \textbf{m}_0) \ \text{ measurable } \text{ in }\ \Omega , \ \mathcal {E}_0 \in [0, \infty ) \Big |\ \int _{\Omega } E(\varrho _0, \textbf{m}_0) \ \,\textrm{d} {x} \le \mathcal {E}_0 \right\} . \end{aligned}$$

As explained in [8], the total energy $\mathcal {E}$, with its initial value $\mathcal {E}_0$, is formally added as a “new” state variable in the definition of dissipative solutions.

**Figure:**
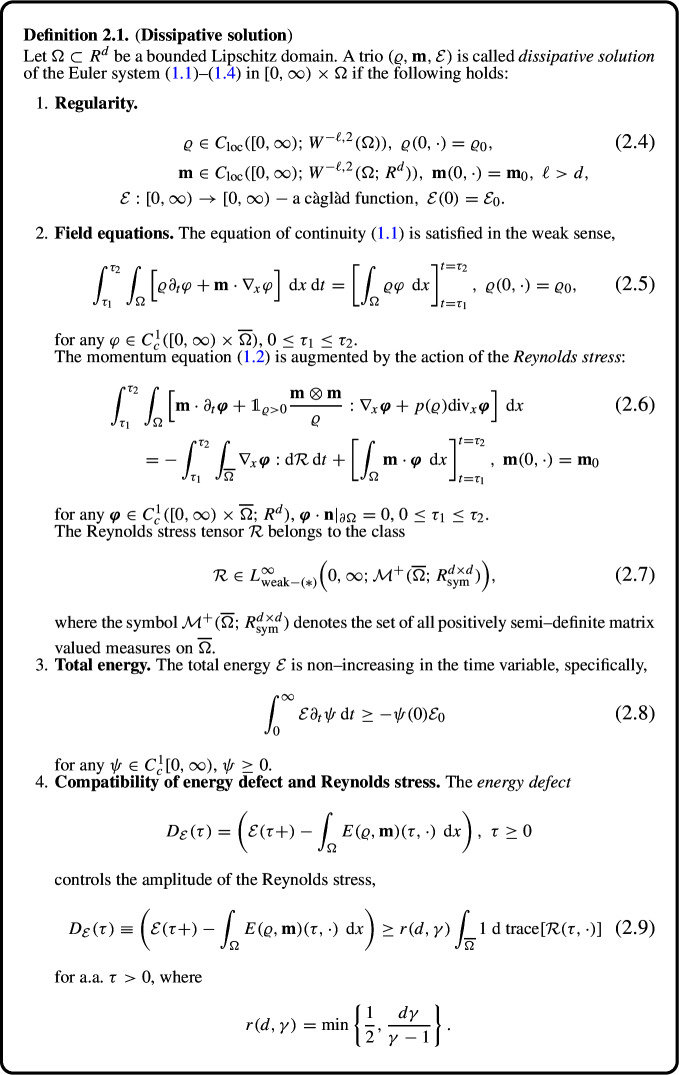

#### Remark 2.2

The specific form of the constant *r* in (2.9) is pertinent to the isentropic equation of state. In the general case, we may consider that

2.10 $$\begin{aligned} \begin{aligned}&p \in C[0, \infty ) \cap C^2(0, \infty ),\ p(0) = 0, \ p'(\varrho )> 0 \ \text{ for }\ \varrho> 0; \\&\text{ the } \text{ pressure } \text{ potential }\ P \ \text{ determined } \text{ by }\ P'(\varrho ) \varrho - P(\varrho ) = p(\varrho )\ \text{ satisfies }\ P(0) = 0,\\  &\text{ and }\ P - {\underline{a}} p,\ \overline{a} p - P\ \text{ are } \text{ convex } \text{ functions } \text{ for } \text{ certain } \text{ constants }\ {\underline{a}}> 0, \ \overline{a} > 0; \end{aligned} \end{aligned}$$

see [1]. The constant *r* in (2.9) is then determined solely in terms of ${\underline{a}}$, $\overline{a}$, and the dimension *d*.

As already pointed out the density—momentum component $(\varrho , \textbf{m})$ of a dissipative solution is nothing other than the expected value of a Young measure for “conventional” measure–valued solutions. The total energy $\mathcal {E}$ is the standard energy $\int _{\Omega } E(\varrho , \textbf{m}) \ \,\textrm{d} {x}$ augmented by the energy defect, where the latter corresponds to the sum of oscillation and concentration defects in the measure–valued formulation.

Note that (2.8), (2.9) imply

2.11 $$\begin{aligned} \varrho (t, \cdot )&\ge 0 \ \text { a.a. } \text { in }\ \Omega ,\ {\textbf {m}}(t, \cdot ) = 0 \ \text { a.a. } \text { on } \text { the } \text { vacuum } \text { set }\ \{ \varrho (t, \cdot ) = 0 \} \ \text { for } t \ge 0, \end{aligned}$$

2.12 $$\begin{aligned} \varrho&\in C_\textrm{weak}([0, \infty ); L^\gamma (\Omega )),\ \textbf{m}\in C_\textrm{weak}([0, \infty ); L^{\frac{2 \gamma }{\gamma + 1}}(\Omega ; R^d)). \end{aligned}$$

#### Rate of Energy Dissipation, Maximal Dissipative Solutions

Let the data $(\varrho _0, \textbf{m}_0, \mathcal {E}_0)$ be given. We denote

$$\begin{aligned}&\mathcal {U}[\varrho _0, \textbf{m}_0, \mathcal {E}_0] = \left\{ (\varrho , \textbf{m}, \mathcal {E})\ \Big |\ (\varrho , \textbf{m}, \mathcal {E}) \ \text{ a } \text{ dissipative } \text{ solution },\ \varrho (0, \cdot ) = \varrho _0,\ \textbf{m}(0, \cdot ) = \textbf{m}_0,\ \mathcal {E}(0)\right. \\&\quad \left. = \mathcal {E}_0 \right\} \end{aligned}$$

as the set of all dissipative solutions on [0,∞) emanating from the data $(\varrho _0, \textbf{m}_0, \mathcal {E}_0)$.

To compare the rate of energy dissipation, we introduce two order relations already discussed in Sect. 1. The first one introduced by DiPerna [18] reads:

2.13 $$\begin{aligned} (\varrho ^1, \textbf{m}^1, \mathcal {E}^1) \prec (\varrho ^2, \textbf{m}^2, \mathcal {E}^2) \ \Leftrightarrow \ \mathcal {E}^1 \le \mathcal {E}^2. \end{aligned}$$

**Figure:**
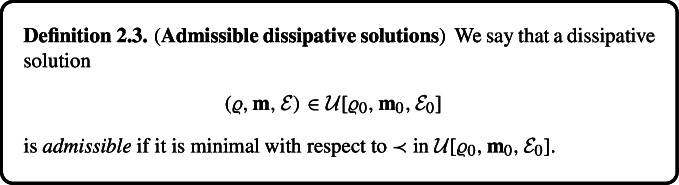

For admissible dissipative solutions, the total energy $\mathcal {E}$ is uniquely determined by $(\varrho , \textbf{m})$. Specifically, the following holds:

##### Proposition 2.4

Let $(\varrho ^i, \textbf{m}^i, \mathcal {E}^i)$ be two admissible solutions emanating from the data $(\varrho ^i_0, \textbf{m}^i_0, \mathcal {E}^i_0)$, i=1,2. Suppose

$$\begin{aligned} (\varrho ^1, {\textbf {m}}^1)(t, \cdot ) = (\varrho ^2, {\textbf {m}}^2)(t, \cdot ) \quad \text {for } \text { any }\ t \in [T, \infty ), \quad T\ge 0. \end{aligned}$$

Then

$$\begin{aligned} \mathcal {E}^1(t) = \mathcal {E}^2(t) \quad \text {for } \text { any }\ t \in [T, \infty ).\end{aligned}$$

##### Proof

Denote that

$$\begin{aligned} \underline{\mathcal {E}} (t) = \min \left\{ \mathcal {E}^1(t), \mathcal {E}^2(t) \right\} ,\ t \in [T, \infty ). \end{aligned}$$

It is easy to check that the solutions

$$\begin{aligned} (\varrho ^i, \textbf{m}^i, \underline{\mathcal {E}}^i),\ i = 1,2, \end{aligned}$$

where

$$\begin{aligned} \underline{\mathcal {E}}^i(t) = \left\{ \begin{array}{ll} \mathcal {E}^i (t), & \ t \in [0,T), \\ \underline{\mathcal {E}}(t), & \ t \in [T, \infty ), \end{array} \right. \quad i=1,2,\end{aligned}$$

are again dissipative solutions with the modified Reynolds stresses

$$\begin{aligned} \underline{\mathcal {R}}^i(t, \cdot ) = \left\{ \begin{array}{ll} \mathbb {1}_{ \mathcal {E}^1(t) > \mathcal {E}^2(t) } \mathcal {R}^2 (t) + \mathbb {1}_{ \mathcal {E}^2(t) \ge \mathcal {E}^2(t) } \mathcal {R}^1(t), & \ t \in [T, \infty ),\\ \mathcal {R}^i(t), & \ t \in [0,T),\\ \end{array} \right. \quad i=1,2. \end{aligned}$$

However, as both solutions are minimal with respect to ≺, we conclude that

$$\begin{aligned} \mathcal {E}^i(t) = \underline{\mathcal {E}}(t),\ t \in [T, \infty ),\ i =1,2. \end{aligned}$$

□

DiPerna’s conjecture stated in [18, Section 6, Part (b)] asserts that “*any admissible dissipative solution is a weak solution*”. To the best of our knowledge this conjecture is still not proven. Moreover, its formulation is not “deterministic” as it depends on the behaviour of solutions on the whole interval (0,∞).

Motivated by Dafermos [14], we introduce a local version of (2.13),

2.14 $$\begin{aligned} (\varrho ^1, \textbf{m}^1, \mathcal {E}^1)&\prec _\textrm{loc} (\varrho ^2, \textbf{m}^2, \mathcal {E}^2)\nonumber \\  &\Leftrightarrow \nonumber \\ \text{ there } \text{ exists }\ T&\ge 0 \ \text{ such } \text{ that }\ (\varrho ^1, \textbf{m}^1, \mathcal {E}^1)(\tau , \cdot ) = (\varrho ^2, \textbf{m}^2, \mathcal {E}^2)(\tau , \cdot ) \ \text{ for } \text{ any }\ \tau \in [0,T],\nonumber \\ \mathcal {E}^1(\tau )&< \mathcal {E}^2(\tau ) \ \text{ for }\ \tau \in (T, T+ \delta ) \ \text{ for } \text{ some }\ \delta > 0. \end{aligned}$$

**Figure:**
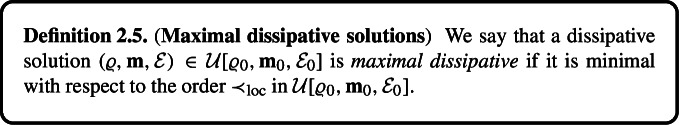

One of the main results of this paper—Theorem 3.3—is a rigorous proof of DiPerna’s conjecture in Dafermos’ framework. more Specifically, we show that any maximal dissipative solution is necessarily a weak solution of the Euler system.

### Properties of Dissipative Solutions

We start by introducing the necessary function spaces framework. In accordance with (2.9), the instantaneous values of dissipative solutions $(\varrho , \textbf{m}, \mathcal {E})(\tau +, \cdot )$ evaluated at a time $\tau \ge 0$ belong to the convex set

2.15 $$\begin{aligned} \mathcal {D}= \left\{ (\varrho , \textbf{m}, \mathcal {E}) \ \Big | \ \varrho , \textbf{m}\ \text{ measurable } \text{ in }\ \Omega ,\ \ \int _{\Omega } E(\varrho , \textbf{m}) \ \,\textrm{d} {x} \le \mathcal {E} \right\} . \end{aligned}$$

Next, we introduce the trajectory space $\mathcal {T}$, in which solutions live. We consider two different topologies on the trajectory space,

2.16 $$\begin{aligned} \mathcal {T}_\textrm{weak} = C_\textrm{loc}([0, \infty ); W^{-\ell ,2}(\Omega )) \times C_\textrm{loc}([0, \infty ); W^{-\ell ,2}(\Omega ;R^d)) \times L^q_{\omega }[0, \infty ), \end{aligned}$$

and

2.17 $$\begin{aligned} \mathcal {T}_\textrm{strong} = L^q_{\omega }([0, \infty ); L^q(\Omega )) \times L^{q}_{\omega }([0, \infty ); L^{q}(\Omega ;R^d)) \times L^q_{\omega }[0, \infty ), \end{aligned}$$

$1< q \le \frac{2 \gamma }{\gamma + 1} < \gamma$, where $\omega (t) = \exp (-t)$ is an exponential weight function. Note that the weighted Lebesgue measure $\omega \frac{{\textrm{d}}}{{\textrm{d}}t}\otimes \,\textrm{d} {x}$ considered on $[0,\infty ) \times \Omega$ is of finite measure. The space $\mathcal {T}_\textrm{weak}$ is a separable, complete metric space, while $\mathcal {T}_\textrm{strong}$ is a uniformly convex, separable, reflexive Banach space. In particular, both topologies are Polish.

#### Global Existence

Given the data $(\varrho _0, \textbf{m}_0, \mathcal {E}_0) \in \mathcal {D}$ we introduce the solution set

2.18 $$\begin{aligned} \mathcal {U}&[\varrho _0, \textbf{m}_0, \mathcal {E}_0]\nonumber \\  &= \Big \{ (\varrho , \textbf{m}, \mathcal {E}) \ \text{ a } \text{ dissipative } \text{ solution } \text{ with } \text{ the } \text{ data } \ (\varrho _0, \textbf{m}_0, \mathcal {E}_0) \ \text{ defined } \text{ for } \text{ all }\ t \ge 0 \Big \} \nonumber \\  &\subset \mathcal {T}_\textrm{weak} \cap \mathcal {T}_\textrm{strong}. \end{aligned}$$

As shown in [8, Section 3.1], the set $\mathcal {U}[\varrho _0, \textbf{m}_0, \mathcal {E}_0]$ is non-empty, meaning the dissipative solution exists for any data $(\varrho _0, \textbf{m}_0, \mathcal {E}_0) \in \mathcal {D}$.

##### Remark 2.6

As a matter of fact, the existence in [8] was established for periodic boundary conditions. The extension to the impermeability conditions is, however, straightforward (see also [7]).

#### Convexity of the Solution Set

We start with a crucial observation.

##### Lemma 2.7

(**Convexity**) For any initial data $(\varrho _0, \textbf{m}_0, \mathcal {E}_0) \in \mathcal {D}$, the solution set $\mathcal {U}[\varrho _0, \textbf{m}_0, \mathcal {E}_0]$ is convex.

##### Proof

Let

$$\begin{aligned} (\varrho , {\textbf {m}}, \mathcal {E}) = \lambda (\varrho ^1, {\textbf {m}}^1, \mathcal {E}^1) + (1 - \lambda ) (\varrho ^2, {\textbf {m}}^2, \mathcal {E}^2),\quad \lambda \in [0,1], \end{aligned}$$

where

$$\begin{aligned} (\varrho ^i, {\textbf {m}}^i, \mathcal {E}^i) \in \mathcal {U}[\varrho _0, {\textbf {m}}_0, \mathcal {E}_0],\quad i =1,2.\end{aligned}$$

Obviously, the trio $(\varrho , \textbf{m}, \mathcal {E})$ belongs to the regularity class (2.4), and the equation of continuity (2.5) as well as the energy inequality (2.8) are satisfied.

As for the momentum balance (2.6), we easily deduce

$$\begin{aligned}&\int _0^\tau \int _{\Omega } \Big [ {\textbf {m}}\cdot \partial \boldsymbol{\varphi }+ \mathbb {1}_{\varrho > 0} \frac{{\textbf {m}}\otimes {\textbf {m}}}{\varrho }: \nabla _x\boldsymbol{\varphi }+ p(\varrho ) \text {div}_x\boldsymbol{\varphi }\Big ] \ \,\text {d} {x}\\  &\quad = - \int _0^\tau \int _{\overline{\Omega }} \nabla _x\boldsymbol{\varphi }: \text {d}{\mathcal {R}} \,\text {d} t + \left[ \int _{\Omega } {\textbf {m}}\cdot \boldsymbol{\varphi } \ \,\text {d} {x} \right] _{t = 0}^{t = \tau } \end{aligned}$$

for any $\boldsymbol{\varphi }\in C^1_c([0, \infty ) \times \overline{\Omega }; R^d)$, $\boldsymbol{\varphi }\cdot \textbf{n}|_{\partial \Omega } = 0$, with a new Reynolds stress

2.19 $$\begin{aligned} \mathcal {R}&= \lambda \mathcal {R}^1 + (1 - \lambda ) \mathcal {R}^2 + \lambda \mathbb {1}_{\varrho ^1> 0} \frac{\textbf{m}^1 \otimes \textbf{m}^1}{\varrho ^1} + (1 - \lambda ) \mathbb {1}_{\varrho ^2> 0} \frac{\textbf{m}^2 \otimes \textbf{m}^2}{\varrho ^2} \nonumber \\&\quad - \mathbb {1}_{[\lambda \varrho _1 + (1 - \lambda ) \varrho _2]>0} \frac{(\lambda \textbf{m}^1 + (1 - \lambda ) \textbf{m}^2 ) \otimes (\lambda \textbf{m}^1 + (1 - \lambda ) \textbf{m}^2 ) }{\lambda \varrho ^1 + (1 - \lambda ) \varrho ^2} \nonumber \\  &\quad + \left[ \Big ( \lambda p(\varrho ^1) + (1 - \lambda ) p(\varrho ^2) \Big ) - p \left( \lambda \varrho ^1 + (1 - \lambda ) \varrho ^2 \right) \right] \mathbb {I}. \end{aligned}$$

It is easy to check

$$\begin{aligned}&\left[ \lambda \mathbb {1}_{\varrho ^1> 0} \frac{\textbf{m}^1 \otimes \textbf{m}^1}{\varrho ^1} + (1 - \lambda ) \mathbb {1}_{\varrho ^2> 0} \frac{\textbf{m}^2 \otimes \textbf{m}^2}{\varrho ^2} \right. \\&\qquad \left. - \mathbb {1}_{\lambda \varrho _1 + (1 - \lambda ) \varrho _2> 0} \frac{(\lambda \textbf{m}^1 + (1 - \lambda ) \textbf{m}^2 ) \otimes (\lambda \textbf{m}^1 + (1 - \lambda ) \textbf{m}^2 ) }{[\lambda \varrho ^1 + (1 - \lambda ) \varrho ^2]> 0} \right] : (\xi \otimes \xi ) \\&\quad = \lambda \mathbb {1}_{\varrho ^1> 0} \frac{|\textbf{m}^1 \cdot \xi |^2}{\varrho ^1} + (1 - \lambda ) \mathbb {1}_{\varrho ^2> 0} \frac{|\textbf{m}^2 \cdot \xi |^2}{\varrho ^2} - \mathbb {1}_{[\lambda \varrho ^1 + (1 - \lambda ) \varrho ^2]> 0} \frac{|(\lambda \textbf{m}^1 + (1 - \lambda ) \textbf{m}^2 )) \cdot \xi |^2 }{\lambda \varrho ^1 + (1 - \lambda ) \varrho ^2} \end{aligned}$$

Using the fact that $\textbf{m}_i = 0$ whenever $\varrho _i = 0$, convexity of the function

$$\begin{aligned} (\varrho , \textbf{m}) \mapsto \frac{|\textbf{m}\cdot \xi |^2}{\varrho }, \end{aligned}$$

and convexity of *p*, we conclude that the new Reynolds stress is positively semi–definite, meaning (2.7) holds.

Finally, by the same token,

$$\begin{aligned}  &   \mathcal {E} {-} \int _{\Omega } E(\varrho , \textbf{m}) \ \,\textrm{d} {x} {=} \lambda \mathcal {E}^1 {-} \lambda \int _{\Omega } E(\varrho ^1, \textbf{m}^1) \ \,\textrm{d} {x} {+} (1 {-} \lambda ) \mathcal {E}^2 {-} (1 {-} \lambda ) \int _{\Omega } E(\varrho ^2, \textbf{m}^2) \ \,\textrm{d} {x}\\  &   {+} \lambda \int _{\Omega } E(\varrho ^1, \textbf{m}^1) \ \,\textrm{d} {x} {+} (1 {-} \lambda ) \int _{\Omega } E(\varrho ^2, \textbf{m}^2) \ \,\textrm{d} {x} {-} \int _{\Omega } E\left( \lambda \varrho ^1 {+} (1 {-} \lambda ) \varrho ^2),\right. \left. \lambda \textbf{m}^1 + (1 - \lambda )\textbf{m}^2 \right) \ \,\textrm{d} {x} \\  &   \ge \lambda r(d,\gamma ) \int _{\overline{\Omega }} \textrm{d}\ \textrm{trace}\ [\mathcal {R}^1] \,\textrm{d} {x}+ (1 - \lambda ) r(d,\gamma ) \int _{\overline{\Omega }} \textrm{d}\ \textrm{trace} \ [\mathcal {R}^2] \,\textrm{d} {x}\\  &   {+}\lambda \int _{\Omega } E(\varrho ^1, \textbf{m}^1) \ \,\textrm{d} {x} {+} (1 {-} \lambda ) \int _{\Omega } E(\varrho ^2, \textbf{m}^2) \ \,\textrm{d} {x} {-} \int _{\Omega } E\Big ( \lambda \varrho ^1 {+} (1 {-} \lambda ) \varrho ^2), \lambda \textbf{m}^1 {+} (1 {-} \lambda )\textbf{m}^2 \Big ) \ \,\textrm{d} {x}\\  &   \ge r(d, \gamma ) \int _{\overline{\Omega }} \ \textrm{d}\ \textrm{trace}[\mathcal {R}], \end{aligned}$$

which yields the compatibility condition (2.9). □

Summarizing, we may infer that the solution set $\mathcal {U}[\varrho _0, \textbf{m}_0, \mathcal {E}_0]$ is:

- non–empty, convex, compact in $\mathcal {T}_\textrm{weak}$;
- non–empty, convex, closed and bounded in $\mathcal {T}_\textrm{strong}$.

#### Topological Properties of the Solution Set

It is convenient to consider the data space $\mathcal {D}$ endowed with the (strong) topology of the Hilbert space

2.20 $$\begin{aligned} \mathcal {D} \subset W^{-\ell ,2}(\Omega ) \times W^{-\ell ,2}(\Omega ; R^d) \times R,\ \ell > d. \end{aligned}$$

More specifically, the data space $\mathcal {D}$ is a convex, locally compact subset of the aforementioned Hilbert space.

We recall the following result proved in [8, Section 3.1].

##### Proposition 2.8

(**Sequential stability**) Consider a sequence of data

$$\begin{aligned} (\varrho _{0,n}, {\textbf {m}}_{0,n}, \mathcal {E}_{0,n}) \rightarrow (\varrho _0, {\textbf {m}}_0, \mathcal {E}_0) \quad \text {in }\ \mathcal {D}\ \text { as }\ n \rightarrow \infty .\end{aligned}$$

Let

$$\begin{aligned} (\varrho _n, {\textbf {m}}_n, \mathcal {E}_n) \in \mathcal {U} [\varrho _{0,n}, {\textbf {m}}_{0,n}, \mathcal {E}_{0,n}] \quad \text {for }\ n =1,2,\dots . \end{aligned}$$

Then there is a subsequence such that

$$\begin{aligned} (\varrho _{n_k}, {\textbf {m}}_{n_k}, \mathcal {E}_{n_k}) \rightarrow (\varrho , {\textbf {m}}, \mathcal {E}) \quad \text {in }\ \mathcal {T}_\text {weak} \ \text { as }\ k \rightarrow \infty , \end{aligned}$$

where

$$\begin{aligned} (\varrho , \textbf{m}, \mathcal {E}) \in \mathcal {U}[\varrho _0, \textbf{m}_0, \mathcal {E}_0]. \end{aligned}$$

The sequential stability yields, in particular, the closedness of the graph of the multivalued solution mapping that is crucial for Borel measurability of the latter with respect to the data discussed in the next section.

#### Measurability of the Solution Set in $\mathcal {T}_\textrm{weak}$

We recall that a set–valued mapping $\mathcal {U}:\mathcal {D} \rightarrow 2^\mathcal {T}$ ranging in a family of subsets of a topological space $\mathcal {T}$ is (weakly) measurable if the set

$$\begin{aligned} \left\{ d \in \mathcal {D} \ \Big | \ \mathcal {U}(d) \cap B \ne \emptyset \right\} \end{aligned}$$

are measurable for any open set $B \subset \mathcal {T}$. It is worth noting that $\mathcal {U}$ is measurable if and only if $\textrm{cl}[\mathcal {U}]$ is measurable. If $\mathcal {D}$ is a topological space, we may define Borel measurability requiring the set to be Borel measurable.

As $\mathcal {T}_\textrm{weak}$ is a separable complete metric space and the sets $\mathcal {U}$ are compact, (Borel) measurability is equivalent to (Borel) measurability of the mapping

$$\begin{aligned} \mathcal {U}: \mathcal {D} \rightarrow \textrm{comp}[\mathcal {T}_\textrm{weak}], \end{aligned}$$

where $\textrm{comp}[\mathcal {T}_\textrm{weak}]$ is the metric space of all compact subsets of $\mathcal {T}_\textrm{weak}$ endowed with the Hausdorff topology.

As shown in [8, Section 3.1], the solution mapping

2.21 $$\begin{aligned} \mathcal {U}: \mathcal {D} \subset W^{-\ell ,2}(\Omega ) \times W^{-\ell ,2}(\Omega ; R^d) \times R \rightarrow \textrm{comp}[ \mathcal {T}_\textrm{weak}] \end{aligned}$$

is Borel measurable.

#### Measurability of the Solution Set in $\mathcal {T}_\textrm{strong}$

As the solution mapping is Borel measurable with values in compact subsets of $\mathcal {T}_\textrm{weak}$, it admits the so–called Castaign representation—a countable family of Borel measurable selections

$$\begin{aligned} U_i: \mathcal {D} \rightarrow \mathcal {T}_\text {weak},\ U_i (d) \in \mathcal {U}_i [d] \quad \text {for } \text { any }\ d \in \mathcal {D},\ i=1,2,\dots , \end{aligned}$$

such that

$$\begin{aligned} \text {cl}_{\mathcal {T}_\text {weak}}[ (U_i(d))_{i=1}^\infty ] = \mathcal {U}[d] \quad \text {for } \text { any }\ d \in \mathcal {D}. \end{aligned}$$

Since the sets $\mathcal {U}$ are convex, we may extend the family $U_i$ by considering convex combinations

$$\begin{aligned} \sum _{j=1}^m \lambda _j U_i,\quad \lambda _j \in Q^+,\quad \sum _{j=1}^m \lambda _j = 1 \end{aligned}$$

with rational coefficients. As the closures of convex sets in the weak and strong topology coincide, we infer that the augmented system is a Castaign representation with respect to the topology $\mathcal {T}_\textrm{strong}$. Thus

2.22 $$\begin{aligned} \mathcal {U}: \mathcal {D} \subset W^{-\ell ,2}(\Omega ) \times W^{-\ell ,2}(\Omega ; R^d) \times R \rightarrow \textrm{closed}[ \mathcal {T}_\textrm{strong}] \end{aligned}$$

is Borel measurable.

Finally, by Hess’ measurability theorem [27], the measurability stated in (2.22) is equivalent to the Borel measurability of the set valued mapping

$$\begin{aligned} \mathcal {U}: \mathcal {D} \rightarrow \textrm{closed}[{\mathcal {T}}_\textrm{strong}], \end{aligned}$$

where $\textrm{closed}[\mathcal {T}_\textrm{strong}]$ is the set of all closed subsets of $\mathcal {T}_\textrm{strong}$ endowed with the (metrizable) Wijsman topology:

2.23 $$\begin{aligned} \mathcal {A}_n {\mathop {\rightarrow }\limits ^{\mathcal {W}}}\mathcal {A} \ \Leftrightarrow \ \textrm{dist}_{\mathcal {T}_\textrm{strong}}[y, \mathcal {A}_n] \rightarrow \textrm{dist}_{\mathcal {T}_\textrm{strong}}[y, \mathcal {A}] \ \text{ for } \text{ any }\ y \in \mathcal {T}_\textrm{strong}. \end{aligned}$$

## Regularity of Dissipative Solutions

We are ready to state and prove our main results concerning regularity of dissipative solutions. They include, in particular:

- The existence of dissipative solutions with arbitrarily small Reynolds stress (energy defect).
- Regularity of maximal dissipative solutions, namely any dissipative solution minimal with respect to $\prec _\textrm{loc}$ is an admissible weak solution.

### Dissipative Solutions with Small Energy Defect

We show the existence of dissipative solutions with arbitrarily small energy defect

$$\begin{aligned} {D}_{\mathcal {E}}(\tau ) = \mathcal {E}(\tau +) - \int _{\Omega } E(\varrho , \textbf{m})(\tau , \cdot ) \ \,\textrm{d} {x}. \end{aligned}$$

In view of the compatibility relation (2.9), smallness of $D_{\mathcal {E}}$ entails smallness of the norm of the Reynolds stress $\mathcal {R}$.

The result follows from two crucial properties of dissipative solutions:

- **Initial time regularity.** Suppose that
  3.1 $$\begin{aligned} \mathcal {E}(\tau +) = \int _{\Omega } E(\varrho , {\textbf {m}}) (\tau ) \ \,\text {d} {x} \ \Leftrightarrow \ D_{\mathcal {E}}(\tau +) = 0 \quad \text {for } \text { some }\ \tau \ge 0.\end{aligned}$$
  Then for any δ>0 there exists T=T(δ)>0 such that
  3.2 $$\begin{aligned} 0 \le {D}_{\mathcal {E}}(t) \le \delta \quad \text {for } \text { any }\ \tau \le t < \tau + T(\delta ). \end{aligned}$$
  Indeed, as the energy is weakly l.s.c. and the total energy $\mathcal {E}$ non–increasing, we have
  $$\begin{aligned} 0 \le \limsup _{t \rightarrow \tau +} {D}_{\mathcal {E}} (t)&\le \limsup _{t \rightarrow \tau +} \mathcal {E}(t+) - \liminf _{t \rightarrow \tau } \int _{\Omega } E(\varrho , \textbf{m})(t, \cdot ) \ \,\textrm{d} {x} \\  &\le \mathcal {E}( \tau +) - \int _{\Omega } E(\varrho , \textbf{m})(\tau , \cdot ) \ \,\textrm{d} {x} = 0. \end{aligned}$$
- **Concatenation.** Suppose $$(\varrho ^1, \textbf{m}^1, \mathcal {E}^1) \in \mathcal {U}[\varrho _0, \textbf{m}_0, \mathcal {E}_0]$$ and $$(\varrho ^2, \textbf{m}^2, {\mathcal {E}^2}) \in \mathcal {U}[\varrho (T, \cdot ), \textbf{m}(T, \cdot ),$$$$ \mathcal {E}^2_T]$$,
  $$\begin{aligned} \int _{\Omega } E(\varrho , \textbf{m})(T, \cdot ) \ \,\textrm{d} {x} \le \mathcal {E}^2_T \le \mathcal {E}^1(T). \end{aligned}$$
  Then
  $$\begin{aligned} (\varrho , {\textbf {m}}, \mathcal {E})(t, \cdot ) = \left\{ \begin{array}{ll}(\varrho ^1, {\textbf {m}}^1, \mathcal {E}^1)(t, \cdot ) & \ \text { for }\ 0 \le t \le T, \\ (\varrho ^2, {\textbf {m}}^2, \mathcal {E}^2)(t - T, \cdot ) & \ \text { for }\ t > T, \end{array} \right. \end{aligned}$$
  belongs to the the solution set $\mathcal {U}[\varrho _0, \textbf{m}_0, \mathcal {E}_0]$.

Our main result stated below is based on an application of Zorn’s lemma equivalent to the axiom of choice.

**Figure:**
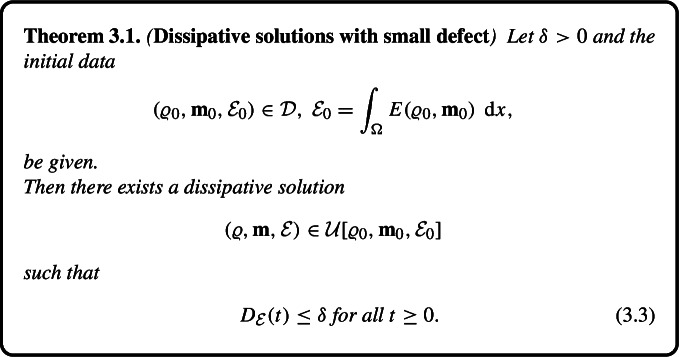

#### Proof

For each global solution

$$\begin{aligned} (\varrho , \textbf{m}, \mathcal {E}) \in \mathcal {U}[\varrho _0, \textbf{m}_0, \mathcal {E}_0] \end{aligned}$$

we define a “stopping” time

$$\begin{aligned} T(\delta )[\varrho , \textbf{m}, \mathcal {E}] = \sup \left\{ \tau \ge 0 \ \Big |\ D_{\mathcal {E}} (t) \le \delta \ \text{ for } \text{ any }\ 0 \le t < \tau \right\} . \end{aligned}$$

We know from (3.2) that T(δ)>0. Moreover,

3.4 $$\begin{aligned} D_{\mathcal {E}}(t) \le \delta \quad \text {for } \text { any }\ 0 \le t < T(\delta ). \end{aligned}$$

Next, we introduce the order relation

3.5 $$\begin{aligned} (\varrho ^1, {\textbf {m}}^1, \mathcal {E}^1)&\prec \hspace{-0.2cm} \prec (\varrho ^2, {\textbf {m}}^2, \mathcal {E}^2) \nonumber \\  &\Leftrightarrow \nonumber \\  &\text { Either}\quad (\varrho ^1, {\textbf {m}}^1, \mathcal {E}^1) = (\varrho ^2, {\textbf {m}}^2, \mathcal {E}^2),\nonumber \\  &\text { or}\quad \mathbb {T}(\delta )[\varrho ^1, {\textbf {m}}^1, \mathcal {E}^1]< \infty ,\ T(\delta )[\varrho ^1, {\textbf {m}}^1, \mathcal {E}^1]< T(\delta ) [\varrho ^2, {\textbf {m}}^2, \mathcal {E}^2], \nonumber \\  &\text { and}\quad (\varrho ^1, {\textbf {m}}^1, \mathcal {E}^1)(t) = (\varrho ^2, {\textbf {m}}^2, \mathcal {E}^2)(t) \ \text { for } \text { any }\ 0 \le t < T(\delta )[\varrho ^1, {\textbf {m}}^1, \mathcal {E}^1]. \end{aligned}$$

It is a routine matter to check that $\mathcal {U}[\varrho _0, \textbf{m}_0, \mathcal {E}_0]$ endowed with the relation $\prec \hspace{-0.2cm} \prec$ is a partially ordered set eligible for the application of Zorn’s lemma. Strictly speaking, Zorn’s lemma is applied to the equivalence classes

$$\begin{aligned} (\varrho ^1, \textbf{m}^1, \mathcal {E}^1 )\sim &   (\varrho ^2, \textbf{m}^2, \mathcal {E}^2 ) \\&\Leftrightarrow _\mathrm{{def}}&\\ T(\delta )[\varrho ^1, \textbf{m}^1, \mathcal {E}^1]= &   T(\delta )[\varrho ^2, \textbf{m}^2, \mathcal {E}^2] \ \text{ and }\ (\varrho ^1, \textbf{m}^1, \mathcal {E}^1 )(t,\cdot ) = (\varrho ^2, \textbf{m}^2, \mathcal {E}^2 )(t, \cdot )\\ \text{ for }\ 0&\le t \le T(\delta ). \end{aligned}$$

Let

$$\begin{aligned}  &   (\varrho ^1, \textbf{m}^1, \mathcal {E}^1) \prec \hspace{-0.2cm} \prec (\varrho ^2, \textbf{m}^2, \mathcal {E}^2) \prec \hspace{-0.2cm} \prec \dots \prec \hspace{-0.2cm} \prec (\varrho ^n, \textbf{m}^n, \mathcal {E}^n),\ (\varrho ^i, \textbf{m}^i, \mathcal {E}^i) \in \mathcal {U}[\varrho _0, \textbf{m}_0, \mathcal {E}_0] \ \\  &   \quad \text{ for }\ i=1,2, \dots \end{aligned}$$

be an ordered chain. Without loss of generality, we may assume $(\varrho ^i, \textbf{m}^i, \mathcal {E}^i) \ne (\varrho ^j, \textbf{m}^j, \mathcal {E}^j)$ for i≠j. As the solution set is compact in $\mathcal {T}_\textrm{weak}$, the sequence $(\varrho ^n, \textbf{m}^n, \mathcal {E}^n)_{n=1}^\infty$ contains a converging subsequence (not relabeled)

$$\begin{aligned} (\varrho ^n, {\textbf {m}}^n, \mathcal {E}^n) \rightarrow (\widetilde{\varrho }, \widetilde{{\textbf {m}}}, \widetilde{\mathcal {E}}) \in \mathcal {U}[\varrho _0, {\textbf {m}}_0, \mathcal {E}_0] \quad \text {as }\ n \rightarrow \infty \ \text { in } \ \mathcal {T}_\text {weak}.\end{aligned}$$

In accordance with (3.5),

$$\begin{aligned} (\widetilde{\varrho }, \widetilde{{\textbf {m}}}, \widetilde{ \mathcal {E}})(t) = (\varrho ^n, {\textbf {m}}^n, \mathcal {E}^n)(t) \quad \text {for }\ t < T(\delta )[\varrho ^n, {\textbf {m}}^n, \mathcal {E}^n] \ \text { for } \text { any }\ n = 1,2,\dots .\end{aligned}$$

Now, either $T(\delta )(\widetilde{\varrho }, \widetilde{\textbf{m}}, \widetilde{\mathcal {E}}) = \infty$, meaning $(\widetilde{\varrho }, \widetilde{\textbf{m}}, \widetilde{ \mathcal {E}})$ is an upper bound for the chain, or $T(\delta )[\widetilde{\varrho }, \widetilde{\textbf{m}}, \widetilde{\mathcal {E}}]= {\widetilde{T}} < \infty$. In the latter case, we consider a concatenation

$$\begin{aligned} (\varrho , {\textbf {m}}, \mathcal {E})(t, \cdot ) = \left\{ \begin{array}{ll}(\widetilde{\varrho }, \widetilde{{\textbf {m}}}, \widetilde{\mathcal {E}})(t, \cdot ) & \ \text {for }\ 0 \le t \le {\widetilde{T}}, \\ (\hat{\varrho }, \hat{{\textbf {m}}}, \hat{\mathcal {E}})(t - {\widetilde{T}}, \cdot ) & \ \text {for }\ t > {\widetilde{T}}, \end{array} \right. \end{aligned}$$

where

$$\begin{aligned} (\hat{\varrho }, \hat{\textbf{m}}, \hat{\mathcal {E}}) \in \mathcal {U}\Big (\widetilde{\varrho }({\widetilde{T}}, \cdot ), \widetilde{\textbf{m}}({\widetilde{T}}, \cdot ), \int _{\Omega } E(\widetilde{\varrho }({\widetilde{T}}, \cdot ), \widetilde{\textbf{m}}({\widetilde{T}}, \cdot ) ) \ \,\textrm{d} {x} \Big ). \end{aligned}$$

Applying (3.1), (3.2) to $(\hat{\varrho }, \hat{\textbf{m}}, \hat{\mathcal {E}})$ we can see $({\varrho }, {\textbf{m}}, {\mathcal {E}})$ is an upper bound for the chain. Thus in both cases, the ordered chain admits an upper bound in $\mathcal {U}[\varrho _0, \textbf{m}_0, \mathcal {E}_0]$.

By Zorn’s lemma, there exists a maximal solution $(\widetilde{\varrho }, \widetilde{\textbf{m}}, \widetilde{\mathcal {E}}) \in \mathcal {U}[\varrho _0, \textbf{m}_0, \mathcal {E}_0]$ with respect to the relation $\prec \hspace{-0.2cm} \prec$. We claim that

$$\begin{aligned} T(\delta ) [\widetilde{\varrho }, \widetilde{\textbf{m}}, \widetilde{\mathcal {E}}] = \infty , \end{aligned}$$

which completes the proof. Indeed, if

$$\begin{aligned} T(\delta ) [\widetilde{\varrho }, \widetilde{\textbf{m}}, \widetilde{\mathcal {E}}] = {\widetilde{T}} < \infty , \end{aligned}$$

we could repeat the above argument concatenating the solution $(\widetilde{\varrho }, \widetilde{\textbf{m}}, \widetilde{\mathcal {E}})$ with another solution starting from the initial data

$$\begin{aligned} (\hat{\varrho }, \hat{\textbf{m}}, \hat{\mathcal {E}}) \in \mathcal {U}\Big (\widetilde{\varrho }({\widetilde{T}}, \cdot ), \widetilde{\textbf{m}}({\widetilde{T}}, \cdot ), \int _{\Omega } E(\widetilde{\varrho }({\widetilde{T}}, \cdot ), \widetilde{\textbf{m}}({\widetilde{T}}, \cdot ) ) \ \,\textrm{d} {x} \Big ), \end{aligned}$$

$$\begin{aligned} (\varrho , \textbf{m}, \mathcal {E})(t, \cdot ) = \left\{ \begin{array}{ll}(\widetilde{\varrho }, \widetilde{\textbf{m}}, \widetilde{\mathcal {E}})(t, \cdot ) &  \quad \text{ for }\ 0 \le t \le {\widetilde{T}}, \\ \\ (\hat{\varrho }, \hat{\textbf{m}}, \hat{\mathcal {E}})(t - {\widetilde{T}}, \cdot ) &  \quad \text{ for }\ t > {\widetilde{T}}. \end{array} \right. \end{aligned}$$

Obviously, the new solution $(\varrho , \textbf{m}, \mathcal {E})$ satisfies

$$\begin{aligned} (\widetilde{\varrho }, \widetilde{\textbf{m}}, \widetilde{\mathcal {E}}) \prec \hspace{-0.2cm} \prec (\varrho , \textbf{m}, \mathcal {E}), \end{aligned}$$

and, by virtue of the initial regularity property,

$$\begin{aligned} T(\delta ) [{\varrho }, \textbf{m}, \mathcal {E}] \ge {\widetilde{T}} + T(\delta ) [\hat{\varrho }, \hat{\textbf{m}}, \hat{\mathcal {E}}] > {\widetilde{T}}. \end{aligned}$$

Consequently, $(\widetilde{\varrho }, \widetilde{\textbf{m}}, \widetilde{\mathcal {E}})$ is not maximal which is a contradiction.

□

Applying the same approach we can show the existence of a dissipative solution with the energy defect dominated by an arbitrary positive function, in particular, the defect may vanish for $t \rightarrow \infty$.

**Figure:**
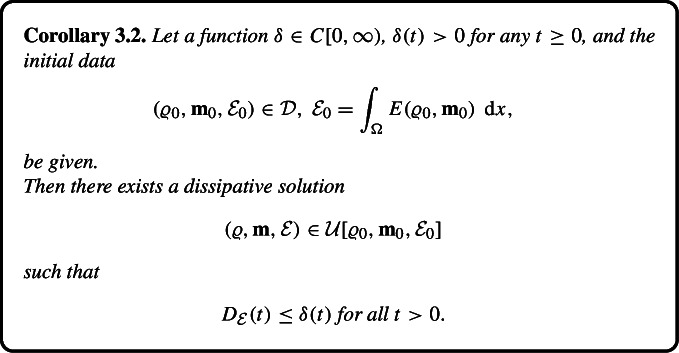

#### Proof

Apply the arguments of the proof of Theorem 3.1 to obtain a solution $(\varrho , \textbf{m}, \mathcal {E})$, with the energy defect

$$\begin{aligned} D_{\mathcal {E}}(t) \le \min _{\tau \in [n, n+1]} \delta (\tau ) \quad \text {for }\ t \in [n, n+1].\end{aligned}$$

□

### Maximal Dissipation Principle

We recall the order relation introduced in (2.14), namely

$$\begin{aligned} (\varrho ^1, \textbf{m}^1, \mathcal {E}^1)&\prec _\textrm{loc} (\varrho ^2, \textbf{m}^2, \mathcal {E}^2) \\  &\Leftrightarrow \\ \text{ there } \text{ exists }\ T&\ge 0 \ \text{ such } \text{ that }\ (\varrho ^1, \textbf{m}^1, \mathcal {E}^1)(\tau , \cdot ) = (\varrho ^2, \textbf{m}^2, \mathcal {E}^2)(\tau , \cdot ) \ \text{ for } \text{ any }\ \\ \tau \in [0,T], \mathcal {E}^1(\tau )&< \mathcal {E}^2(\tau ) \ \text{ for }\ \tau \in (T, T+ \delta ) \ \text{ for } \text{ some }\ \delta > 0. \end{aligned}$$

Similarly to the relation $\prec \hspace{-0.2cm} \prec$ introduced in the proof of Theorem 3.1, the order relation $\prec _\textrm{loc}$ augmented formally by the identity relation represents a partial ordering of $\mathcal {U}[\varrho _0, \textbf{m}_0, \mathcal {E}_0]$. Recall that a dissipative solution is maximal (dissipative) if it is minimal with respect to $\prec _\textrm{loc}$.

#### Regularity of Maximal Dissipative Solutions

Our next goal is to show that any maximal dissipative solution in the sense of Definition 2.5 is necessarily an admissible (with non–increasing total energy) weak solution. The result can be seen as a rigorous verification of DiPerna’s conjecture [18, Section 6] in the context of the barotropic Euler system.

**Figure:**
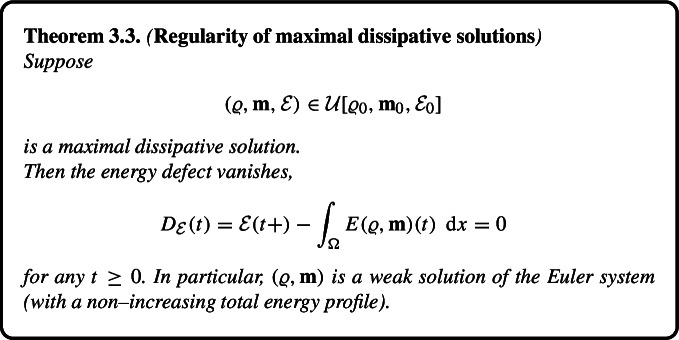

##### Proof

Suppose

$$\begin{aligned} D_{\mathcal {E}}(T) = \mathcal {E}(T+) - \int _{\Omega } E(\varrho , \textbf{m}) (T) \ \,\textrm{d} {x} = \varepsilon > 0 \end{aligned}$$

for some T≥0. As $\mathcal {E}$ is non–increasing, there exists δ>0 such that

3.6 $$\begin{aligned} \mathcal {E}(t) > \mathcal {E}(T+) - \frac{\varepsilon }{2} \quad \text {for } \text { any }\ t \in (T, T + \delta ). \end{aligned}$$

Next, by concatenation, we may construct a new solution of the same problem such that

$$\begin{aligned} (\widetilde{\varrho }, \widetilde{{\textbf {m}}}, \widetilde{\mathcal {E}})(t, \cdot ) = \left\{ \begin{array}{ll}(\varrho , {\textbf {m}}, {\mathcal {E}})(t, \cdot ) & \ \text { for }\ 0 \le t \le T, \\ (\hat{\varrho }, \hat{{\textbf {m}}}, \hat{\mathcal {E}})(t - {\widetilde{T}}, \cdot ) & \ \text { for }\ t > {T}, \end{array} \right. \end{aligned}$$

where

$$\begin{aligned} (\hat{\varrho }, \hat{\textbf{m}}, \hat{\mathcal {E}}) \in \mathcal {U}\Big (\varrho ({T}, \cdot ), \textbf{m}({T}, \cdot ), \int _{\Omega } E(\varrho ({T}, \cdot ), \textbf{m}({T}, \cdot ) ) \ \,\textrm{d} {x} \Big ). \end{aligned}$$

Obviously,

$$\begin{aligned} (\widetilde{\varrho }, \widetilde{{\textbf {m}}}, \widetilde{\mathcal {E}})(t, \cdot ) = (\varrho , {\textbf {m}}, \mathcal {E})(t, \cdot ) \quad \text {for } \text { all }\ t \in [0,T],\end{aligned}$$

while, by virtue of (3.6),

$$\begin{aligned} \widetilde{\mathcal {E}} (t) \le \int _{\Omega } E(\varrho ({T}, \cdot ), \textbf{m}({T}, \cdot )) \ \,\textrm{d} {x} = \mathcal {E}(T+) - \varepsilon < \mathcal {E}(t) \end{aligned}$$

for any $t \in (T, T+ \delta )$. We conclude that $(\varrho , \textbf{m}, \mathcal {E})$ is not minimal.

□

As we shall see in Sect. 4, the existence of admissible dissipative solutions, meaning solutions minimal with respect to the order ≺ is guaranteed for any finite energy data. Unfortunately, however, the existence of maximal dissipative solutions, meaning minimal with respect to the order $\prec _\textrm{loc}$ remains an open problem.

## Semigroup (Semiflow) Selection

Before discussing any selection process, we define two operations on the trajectory space $\mathcal {T}_\textrm{weak}$:

- **Time shift.** For any T≥0 and any $(\varrho , \textbf{m}, \mathcal {E}) \in \mathcal {T}_\textrm{weak}$, we define
  $$\begin{aligned} \mathcal {S}_T[ \varrho , {\textbf {m}}, \mathcal {E} ](t, \cdot ) = (\varrho (t + T), {\textbf {m}}(t +T), \mathcal {E}(t + T) ),\quad t \ge 0. \end{aligned}$$
- **Concatenation.** For
  $$\begin{aligned} (\varrho ^i, \textbf{m}^i, \mathcal {E}^i) \in \mathcal {T}_\textrm{weak}, \ i=1,2,\ T > 0, \end{aligned}$$
  we define that
  $$\begin{aligned} (\varrho ^1, \textbf{m}^1, \mathcal {E}^1) \cup _T (\varrho ^2, \textbf{m}^2, \mathcal {E}^2) = (\varrho , \textbf{m}, \mathcal {E}), \end{aligned}$$
  $$\begin{aligned} (\varrho , \textbf{m}, \mathcal {E}) = \left\{ \begin{array}{ll} (\varrho ^1, \textbf{m}^1, \mathcal {E}^1) (t, \cdot )&  \ \text{ for }\ 0 \le t \le T, \\ \\ (\varrho ^2, \textbf{m}^2, \mathcal {E}^2) (t - T, \cdot )&  \ \text{ for }\ t > T \end{array} \right. \end{aligned}$$
  Note that $(\varrho ^1, \textbf{m}^1, \mathcal {E}^1) \cup _T (\varrho ^2, \textbf{m}^2, \mathcal {E}^2)$ belongs to $\mathcal {T}_\textrm{weak}$, provided that
  $$\begin{aligned} (\varrho ^2(0, \cdot ), \textbf{m}^2(0, \cdot )) = (\varrho ^1(T, \cdot ), \textbf{m}^1(T, \cdot )). \end{aligned}$$

### Properties of the Solution Sets

We recall the basic properties of the solution sets $\mathcal {U}$ established in Sect. 2.2.

- **[A1]** For any data $(\varrho _0, \textbf{m}_0, \mathcal {E}_0) \in \mathcal {D}$, the solution set
  $$\begin{aligned} \mathcal {U} [\varrho _0, \textbf{m}_0, \mathcal {E}_0] \subset \mathcal {T}_\textrm{weak} \cap \mathcal {T}_\textrm{strong} \end{aligned}$$
  is a non–empty convex set, compact in $\mathcal {T}_\textrm{weak}$ and closed bounded in $\mathcal {T}_\textrm{strong}$.
- **[A2]** If
  $$\begin{aligned} (\varrho , \textbf{m}, \mathcal {E}) \in \mathcal {U}[\varrho _0, \textbf{m}_0, \mathcal {E}_0], \end{aligned}$$
  than the time shift satisfies
  $$\begin{aligned} \mathcal {S}_T [\varrho , \textbf{m}, \mathcal {E}] \in \mathcal {U}[ \varrho (T, \cdot ), \textbf{m}(T, \cdot ), \mathcal {E}(T) ] \end{aligned}$$
  for any T≥0.
- **[A3]** If
  $$\begin{aligned} (\varrho ^1, \textbf{m}^1, \mathcal {E}^1) \in \mathcal {U}[\varrho _0, \textbf{m}_0, \mathcal {E}_0] \end{aligned}$$
  and
  $$\begin{aligned}&(\varrho ^2, {\textbf {m}}^2, \mathcal {E}^2) \in \mathcal {U}_{\mathcal {F}}[\varrho ^1(T, \cdot ), {\textbf {m}}^1(T, \cdot ), \mathcal {E}^2_0], \\  &\text { where }\ \int _{\Omega } E(\varrho ^1, {\textbf {m}}^1)(T, \cdot ) \ \,\text {d} {x}\le \mathcal {E}^2_0 \le \mathcal {E}^1(T), \end{aligned}$$
  then
  $$\begin{aligned} (\varrho ^1, \textbf{m}^1, \mathcal {E}^1) \cup _T (\varrho ^2, \textbf{m}^2, \mathcal {E}^2) \in \mathcal {U}[\varrho _0, \textbf{m}_0, \mathcal {E}_0]. \end{aligned}$$
- **[A4]** The set valued mappings
  $$\begin{aligned} (\varrho _0, \textbf{m}_0, \mathcal {E}_0) \in \mathcal {D} \mapsto \mathcal {U}[\varrho _0, \textbf{m}_0, \mathcal {E}_0] \in \textrm{comp}[\mathcal {T}_\textrm{weak}] \end{aligned}$$
  and
  $$\begin{aligned} (\varrho _0, \textbf{m}_0, \mathcal {E}_0) \in \mathcal {D} \mapsto \mathcal {U}[\varrho _0, \textbf{m}_0, \mathcal {E}_0] \in \textrm{closed}[ \mathcal {T}_\textrm{strong}] \end{aligned}$$
  are Borel measurable with respect to the Hausdorff and the Wijsman topology, respectively.

### Selection Process

We consider a functional of the form

$$\begin{aligned} \mathcal {F}(\varrho , \textbf{m}, \mathcal {E}) = \int _0^\infty \exp (-t) F(\varrho (t, \cdot ), \textbf{m}(t, \cdot ), \mathcal {E}(t, \cdot )) \,\textrm{d} t , \end{aligned}$$

where

$$\begin{aligned} F: \mathcal {D} \rightarrow [0, \infty ] \end{aligned}$$

is a convex l.s.c. functional.

For each solution set

$$\begin{aligned} \mathcal {U}[\varrho _0, \textbf{m}_0, \mathcal {E}_0], \end{aligned}$$

let

$$\begin{aligned} \mathcal {U}_{\mathcal {F}}[\varrho _0, \textbf{m}_0, \mathcal {E}_0]&= \left\{ (\widetilde{\varrho }, \widetilde{\textbf{m}}, {\widetilde{E}}) \in \mathcal {U}[\varrho _0, \textbf{m}_0, \mathcal {E}_0] \ \Big | \ \mathcal {F} (\widetilde{\varrho }, \widetilde{\textbf{m}}, \widetilde{\mathcal {E}}) \le \mathcal {F} (\varrho , \textbf{m}, \mathcal {E}) \right. \\&\text{ for } \text{ any }\ (\varrho , \textbf{m}, \mathcal {E}) \in \mathcal {U}[\varrho _0, \textbf{m}_0, \mathcal {E}_0] \Big \} \end{aligned}$$

be the set of minimizers of $\mathcal {F}$ on $\mathcal {U}[\varrho _0, \textbf{m}_0, \mathcal {E}_0]$.

It is easy to see that the restricted solution set $\mathcal {U}_{\mathcal {F}}[\varrho _0, \textbf{m}_0, \mathcal {E}_0]$ is again non–empty and convex. Indeed, suppose $\inf _{\mathcal {U}[\varrho _0, \textbf{m}_0, \mathcal {E}_0]} = I < \infty$, since otherwise the claim is trivial. As the solution set $\mathcal {U}[\varrho _0, \textbf{m}_0, \mathcal {E}_0]$ is compact in $\mathcal {T}_\textrm{weak}$, there is a minimizing sequence $(\varrho _n, \textbf{m}_n, \mathcal {E}_n) \rightarrow (\varrho , \textbf{m}, \mathcal {E}) \in \mathcal {U}[\varrho _0, \textbf{m}_0, \mathcal {E}_0]$ in $\mathcal {T}_\textrm{weak}$. By Fatou’s lemma,

$$\begin{aligned} I&= \lim _{n \rightarrow \infty } \int _0^\infty \exp (-t) F \Big (\varrho _n(t, \cdot ), \textbf{m}_n(t, \cdot ), \mathcal {E}_n(t, \cdot ) \Big ) \,\textrm{d} t \\&\ge \int _0^\infty \exp (-t) \liminf _{n \rightarrow \infty } F \Big (\varrho _n(t, \cdot ), \textbf{m}_n(t, \cdot ), \mathcal {E}_n(t, \cdot ) \Big ) \,\textrm{d} t \\&\ge \int _0^\infty \exp (-t) F \Big (\varrho (t, \cdot ), \textbf{m}(t, \cdot ), \mathcal {E}(t, \cdot ) \Big ) \,\textrm{d} t ; \end{aligned}$$

whence the limit $(\varrho , \textbf{m}, \mathcal {E})$ is a minimizer. The convexity of the set of minimizers follows from the convexity of *F*.

Next, we show that the properties **[A2]**, **[A3]** are satisfied. As a matter of fact, the arguments are almost identical to those of [8, Section 5]. As the present setting is slightly more general, we reproduce the proof for reader’s convenience.

#### Lemma 4.1

- **Shift property.**
  $$\begin{aligned} (\varrho , \textbf{m}, \mathcal {E}) \in \mathcal {U}_{\mathcal {F}}[\varrho _0, \textbf{m}_0, \mathcal {E}_0] \ \Rightarrow \ \mathcal {S}_T (\varrho , \textbf{m}, \mathcal {E}) \in \mathcal {U}_{\mathcal {F}}[ \varrho (T, \cdot ), \textbf{m}(T, \cdot ), \mathcal {E}(T)] \end{aligned}$$
- **Concatenation property.**
  $$\begin{aligned} (\varrho ^1, \textbf{m}^1, \mathcal {E}^1) \in \mathcal {U}_{\mathcal {F}}[\varrho _0, \textbf{m}_0, \mathcal {E}_0] \end{aligned}$$
  and
  $$\begin{aligned}  &   (\varrho ^2, \textbf{m}^2, \mathcal {E}^2) \in \mathcal {U}_{\mathcal {F}}[\varrho ^1(T, \cdot ), \textbf{m}^1(T, \cdot ), \mathcal {E}^2_0], \\  &   \quad \text{ where }\ \int _{\Omega } E(\varrho ^1, \textbf{m}^1)(T, \cdot ) \ \,\textrm{d} {x} \le \mathcal {E}^2_0 \le \mathcal {E}^1(T), \end{aligned}$$
  ⇒
  $$\begin{aligned} (\varrho ^1, \textbf{m}^1, \mathcal {E}^1) \cup _T (\varrho ^2, \textbf{m}^2, \mathcal {E}^2) \in \mathcal {U}_{\mathcal {F}}[\varrho _0, \textbf{m}_0, \mathcal {E}_0]. \end{aligned}$$

#### Proof

The proof is basically the same as in Cardona, Kapintanski [11] or [8]. We present it here for the reader’s convenience.

**Shift property.**

First, by the continuation property,

$$\begin{aligned} (\varrho ^T, \textbf{m}^T, \mathcal {E}^T)\in \mathcal {U}_{\mathcal {F}}[\varrho (T), \textbf{m}(T), \mathcal {E}(T)] \end{aligned}$$

$$\begin{aligned} \Rightarrow \ (\varrho , \textbf{m}, \mathcal {E}) \cup _T[\varrho ^T, \textbf{m}^T, \mathcal {E}^T]\in \mathcal {U}[\varrho _0, \textbf{m}_0, E_0]. \end{aligned}$$

Now

$$\begin{aligned} \mathcal {F}(S_T (\varrho , \textbf{m}, \mathcal {E}))&= \int _0^\infty \exp (-t) F((\varrho , \textbf{m}, \mathcal {E})(t+T)) \,\textrm{d} t \\&= \exp (T) \left( \mathcal {F}((\varrho , \textbf{m}, \mathcal {E})) - \int _0^T \exp (-t) F((\varrho , \textbf{m}, \mathcal {E})(t)) \,\textrm{d} t \right) \\&\le \exp (T) \left( \mathcal {F}((\varrho , \textbf{m}, \mathcal {E}) \cup _T (\varrho ^T, \textbf{m}^T, \mathcal {E}^T)) - \int _0^T \exp (-t) F((\varrho , \textbf{m}, \mathcal {E})(t)) \,\textrm{d} t \right) \\&= \exp (T) \int _T^\infty \exp (-t) F((\varrho ^T, \textbf{m}^T, \mathcal {E}^T)(t - T)) \,\textrm{d} t = \mathcal {F}(\varrho ^T, \textbf{m}^T, \mathcal {E}^T ). \end{aligned}$$

**Concatenation property.**

Compute

$$\begin{aligned}&\mathcal {F}\Big ( (\varrho ^1, \textbf{m}^1, \mathcal {E}^1)\cup _T (\varrho ^2, \textbf{m}^2, \mathcal {E}^2) \Big ) \\&\quad =\int _0^T \exp (-t) F((\varrho ^1, \textbf{m}^1, \mathcal {E}^1)(t))\,\textrm{d} t +\int _T^\infty \exp (-t) F((\varrho ^2, \textbf{m}^2, \mathcal {E}^2)(t-T))\,\textrm{d} t \\&\quad =\int _0^T \exp (-t) F((\varrho ^1, \textbf{m}^1, \mathcal {E}^1)(t))\,\textrm{d} t + \exp (-T) \mathcal {F}(\varrho ^2, \textbf{m}^2, \mathcal {E}^2) \\&\quad \le \int _0^T \exp (-t) F((\varrho ^1, \textbf{m}^1, \mathcal {E}^1)(t))\,\textrm{d} t + \exp (-T) \mathcal {F}(S_T(\varrho ^1, \textbf{m}^1, \mathcal {E}^1)) \\&\quad = \mathcal {F}(\varrho ^1, \textbf{m}^1, \mathcal {E}^1). \end{aligned}$$

□

### First Selection Criterion

The first selection criterion consists in replacing the set

$$\begin{aligned} \mathcal {U}[\varrho _0, \textbf{m}_0, \mathcal {E}_0] \end{aligned}$$

by the set of minimizers of the functional

4.1 $$\begin{aligned} \mathcal {F}_1(\varrho , \textbf{m}, \mathcal {E}) = \int _0^\infty \exp (-t) \mathcal {E}(t) \,\textrm{d} t \end{aligned}$$

considering the topology of the space $\mathcal {T}_\textrm{weak}$. Note that $\mathcal {F}_1(\varrho , \textbf{m}, \mathcal {E})$ is a bounded (continuous) linear form on both $\mathcal {T}_\textrm{weak}$ and $\mathcal {T}_\textrm{strong}$.

Thus we set

4.2 $$\begin{aligned} \mathcal {U}_{\mathcal {F}_1}[\varrho _0, \textbf{m}_0, \mathcal {E}_0]&= \left\{ (\widetilde{\varrho }, \widetilde{\textbf{m}}, \widetilde{\mathcal {E}}) \in \mathcal {U}[\varrho _0, \textbf{m}_0, \mathcal {E}_0] \ \Big | \ \int _0^\infty \exp (-t) \mathcal {E}(t) \,\textrm{d} t \le \int _0^\infty \exp (-t) \widetilde{\mathcal {E}}(t) \,\textrm{d} t \right. \nonumber \\&\left. \quad \quad \text{ for } \text{ all }\ (\widetilde{\varrho }, \widetilde{\textbf{m}}, \widetilde{\mathcal {E}}) \in \mathcal {U}[\varrho _0, \textbf{m}_0, \mathcal {E}_0] \right\} \nonumber \\&\equiv {\arg \min }_{\mathcal {U}[\varrho _0, \textbf{m}_0, \mathcal {E}_0]} \mathcal {F}_1(\varrho , \textbf{m}, \mathcal {E}). \end{aligned}$$

The resulting solution sets are again non–empty, convex and compact in $\mathcal {T}_\textrm{weak}$. Moreover, as shown in [8] the mapping

$$\begin{aligned} \mathcal {A} \subset \mathcal {T}_\textrm{weak} \mapsto \left\{ (\varrho , \textbf{m}, \mathcal {E}) \in \mathcal {A} \ \Big | \ (\varrho , \textbf{m}, \mathcal {E}) = \arg \min _{\mathcal {A}} \mathcal {F}_1(\varrho , \textbf{m}, \mathcal {E}) \subset \mathcal {A} \right\} \end{aligned}$$

is continuous in $\textrm{comp}[\mathcal {T}_\textrm{weak}]$ endowed with the Hausdorff topology.

It is worth noting that we minimize

$$\begin{aligned} \int _0^\infty \exp (-t) \mathcal {E}(t) \,\text {d} t&= \int _0^\infty \exp (-t) \left[ {\mathcal {E}}(t+) - \int _{\Omega } E(\varrho , {\textbf {m}}) (t. \cdot ) \ \,\text {d} {x}\right] \\  &\quad +\int _{\Omega } E(\varrho , {\textbf {m}})(t, \cdot ) \ \,\text {d} {x} \,\text {d} t , \end{aligned}$$

where

$$\begin{aligned} D_{\mathcal {U}}(t) {\mathcal {E}}(t+) - \int _{\Omega } E(\varrho , \textbf{m})(t,\cdot ) \ \,\textrm{d} {x} \ge 0 \end{aligned}$$

is the energy defect, and

$$\begin{aligned} \int _{\Omega } E(\varrho , \textbf{m})(t, \cdot ) \ \,\textrm{d} {x} \end{aligned}$$

the “real” energy of the fluid flow. Moreover, as observed in [8, Section 5], all selected solutions are minimal with respect to the relation ≺, meaning admissible.

### Second Selection Criterion

The next step is to minimize

4.3 $$\begin{aligned} \mathcal {F}_2(\varrho , \textbf{m}, \mathcal {E}) = \int _0^\infty \exp (-t) \left[ \Vert \varrho \Vert ^q_{L^q(\Omega )} + \Vert \textbf{m}\Vert ^q_{L^q(\Omega ; R^d)} + |\mathcal {E}|^q \right] \,\textrm{d} t \end{aligned}$$

on $\mathcal {U}_{\mathcal {F}_1}[\varrho _0, \textbf{m}_0, \mathcal {E}_0]$ for a fixed $1 < q \le 2 \gamma /(\gamma + 1)$. Of course, different exponents *q* may give different minimizers. As the functional is strictly convex, there is a unique minimum attained on any closed convex subset of $\mathcal {T}_\textrm{strong}$. The selected triple $(\varrho , \textbf{m}, \mathcal {E})$ represents a semigroup selection for the compressible Euler system.

In order to show Borel measurability of the data-to-solution mapping, it is enough to show continuity of the mapping

$$\begin{aligned} \mathcal {A} \in \textrm{closed}[\mathcal {T}_\textrm{strong}] \mapsto \arg \min _{\mathcal {A}} F_2 (\varrho , \textbf{m}, \mathcal {E}) \in \mathcal {T}_\textrm{strong} \end{aligned}$$

for $\mathcal {A}$ a closed convex subset belonging $\textrm{closed}[\mathcal {T}_\textrm{strong}]$ endowed with the Wijsman topology.

The space $\mathcal {T}_\textrm{strong}$ is a reflexive, separable, uniformly convex Banach space with a uniformly convex dual. Consequently, the convergence in the Wijsman topology is equivalent to convergence in the Mosco topology $\mathcal {M}$, see [3, Sonntag-Attouch Theorem in Section 4]. We recall that

$$\begin{aligned} \mathcal {A}_n {\mathop {\rightarrow }\limits ^{\mathcal {M}}}\mathcal {A}, \end{aligned}$$

if the two following properties hold:

- For any $(\varrho , \textbf{m}, \mathcal {E}) \in \mathcal {A}$, there exists $(\varrho _n, \textbf{m}_n, \mathcal {E}_n) \in \mathcal {A}_n$ such that
  $$\begin{aligned} (\varrho _n, \textbf{m}_n, \mathcal {E}_n) \rightarrow (\varrho , \textbf{m}, \mathcal {E}) \ \text{ in }\ \mathcal {T}_\textrm{strong}; \end{aligned}$$
- If
  $$\begin{aligned} (\varrho _{n_k}, \textbf{m}_{n_k}, \mathcal {E}_{n_k}) \in \mathcal {A}_{n_k} \rightarrow (\varrho , \textbf{m}, \mathcal {E}) \ \text{ weakly } \text{ in }\ \mathcal {T}_\textrm{strong} \end{aligned}$$
  then
  $$\begin{aligned} (\varrho , \textbf{m}, \mathcal {E}) \in \mathcal {A}. \end{aligned}$$

These conditions guarantee

$$\begin{aligned} \arg \min _{(\varrho , \textbf{m}, \mathcal {E}) \in \mathcal {A}_n} \mathcal {F}_2 (\varrho , \textbf{m}, \mathcal {E}) \rightarrow \arg \min _{(\varrho , \textbf{m}, \mathcal {E}) \in \mathcal {A}} \mathcal {F}_2 (\varrho , \textbf{m}, \mathcal {E}) \end{aligned}$$

whenever

$$\begin{aligned} \mathcal {A}_n {\mathop {\rightarrow }\limits ^{\mathcal {M}}}\mathcal {A},\ \mathcal {A}_n, \mathcal {A} \ \text{ closed } \text{ convex } \text{ subsets } \text{ of }\ \mathcal {T}_\textrm{strong}. \end{aligned}$$

The second selection is therefore Borel measurable mapping from $\mathcal {D}$ to $\mathcal {T}_\textrm{strong}$.

#### Minimizing the Momentum

Alternatively, we can minimize only the momentum in the second step:

4.4 $$\begin{aligned} \mathcal {F}_2(\varrho , \textbf{m}, \mathcal {E}) = \int _0^\infty \exp (-t) \Vert \textbf{m}(t, \cdot ) \Vert ^q_{L^q(\Omega ; R^d)} \ \,\textrm{d} t \end{aligned}$$

considered as a continuous convex functional on $\mathcal {T}_\textrm{strong}$.

The only difference in comparison with the previous section is the fact the

$$\begin{aligned} \arg \min _{\mathcal {A}} \mathcal {F}_2 \end{aligned}$$

is now a set (not a single point) in $\mathcal {T}_\textrm{weak}$. Accordingly, the Borel measurability will follow, as soon as we show that the multivalued mapping

$$\begin{aligned} \arg \min _{\mathcal {A}} \mathcal {F}_2: \textrm{convex}[\mathcal {T}_\textrm{strong}] \rightarrow 2^{\mathcal {T}_\textrm{weak}} \end{aligned}$$

has a closed graph, where the space

$$\begin{aligned} \textrm{convex}[\mathcal {T}_\textrm{strong}] \end{aligned}$$

is endowed with the metrizable Mosco topology.

Suppose

$$\begin{aligned} \mathcal {A}_n {\mathop {\rightarrow }\limits ^{\mathcal {M}}}\mathcal {A} \quad \text {and}\quad (\varrho _n, {\textbf {m}}_n, \mathcal {E}) \in \arg \min _{\mathcal {A}_n} \mathcal {F}_2 \rightarrow (\varrho , {\textbf {m}}, \mathcal {E}) \in \mathcal {T}_\text {weak}. \end{aligned}$$

On the one hand, it follows from convexity and continuity of $\mathcal {F}_2$ that

$$\begin{aligned} \mathcal {F}_2 (\varrho , \textbf{m}, \mathcal {E}) \le \liminf _{n \rightarrow \infty } \mathcal {F}_2 (\varrho _n, \textbf{m}_n, \mathcal {E}_n). \end{aligned}$$

On the other hand, since $\mathcal {A}_n {\mathop {\rightarrow }\limits ^{\mathcal {M}}}\mathcal {A}$, we deduce

$$\begin{aligned} \liminf _{n \rightarrow \infty } \mathcal {F}_2 (\varrho _n, \textbf{m}_n, \mathcal {E}_n) \le \min _{\mathcal {A}} \mathcal {F}_2. \end{aligned}$$

This yields the desired conclusion

$$\begin{aligned} (\varrho , \textbf{m}, \mathcal {E}) \in \arg \min _{\mathcal {A}} \mathcal {F}_2. \end{aligned}$$

Finally, we conclude that this process already gives a unique solution. Indeed, we deduce from the strict convexity of the functional in $\textbf{m}$ that every minimizer has the same momentum component. This in turn implies uniqueness of the associated density as the latter can be recovered from the equation of continuity (2.5). Finally, we know from **step 1** that the selected solution is minimal with respect to ≺. Accordingly, Proposotion 2.4 yields total energy profile $\mathcal {E}$.

### Measurability of the Selected Semigroup

Finally, we realize that the time averages

$$\begin{aligned} \int _0^\infty \theta _{\varepsilon } (\tau - t) \varrho (t, \cdot ) \,\text {d} t , \quad \int _0^\infty \theta _{\varepsilon } (\tau - t) {\textbf {m}}(t, \cdot ) \,\text {d} t ,\end{aligned}$$

where $\theta _\varepsilon$ is a family of regularizing kernels converge weakly

$$\begin{aligned} \int _0^\infty \theta _{\varepsilon } (\tau - t) \varrho (t, \cdot ) \,\text {d} t \rightarrow \varrho (\tau , \cdot ) \quad \text {weakly } \text { in }\ L^\gamma (\Omega ),\end{aligned}$$

$$\begin{aligned} \int _0^\infty \theta _{\varepsilon } (\tau - t) {\textbf {m}}(t, \cdot ) \,\text {d} t \rightarrow {\textbf {m}}(\tau , \cdot )\quad \text {weakly } \text { in }\ L^{\frac{2 \gamma }{\gamma + 1}}(\Omega ; R^d)\end{aligned}$$

as $\varepsilon \rightarrow 0$ for any $\tau \ge$. A Similar argument can be used for $\mathcal {E}(\tau -)$ by means of a “left” regularization. We conclude that the solution mapping

$$\begin{aligned} (\varrho _0, \textbf{m}_0, \mathcal {E}_0) \rightarrow (\varrho (\tau , \cdot ), \textbf{m}(\tau , \cdot ), \mathcal {E}(\tau -)), \end{aligned}$$

being a pointwise limit of Borel measurable mappings, is Borel measurable in $L^{\gamma }(\Omega )-\textrm{weak} \times L^{\frac{2 \gamma }{\gamma +1}}(\mathbb {T}^d; \Omega )-\textrm{weak} \times R$ for any τ>0. Seeing that the weak and strong topology generate the same family of Borel sets, we may also conclude the mapping is strongly measurable.

Let us summarize the results obtained.

**Figure:**
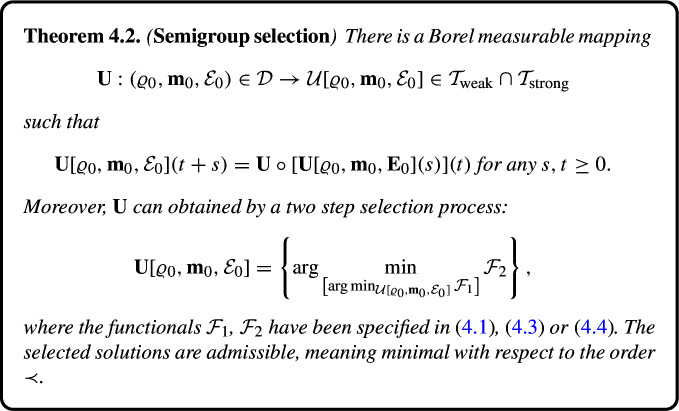

## Absolute Energy Minimizers

Our final goal is to introduce a refined maximal dissipation criterion to recover a unique weak solution. Consider a solution $(\varrho ^1, \textbf{m}^1, \mathcal {E}^1) \in \mathcal {U}[\varrho _0, \textbf{m}_0, \mathcal {E}_0]$ that *is not* maximal. Consequently, there exists $(\varrho ^2, \textbf{m}^2, \mathcal {E}^2) \in \mathcal {U}[\varrho _0, \textbf{m}_0, \mathcal {E}_0]$ such that

$$\begin{aligned} (\varrho ^2, \textbf{m}^2, \mathcal {E}^2) \prec _\textrm{loc} (\varrho ^1, \textbf{m}^1, \mathcal {E}^1), \end{aligned}$$

meaning

$$\begin{aligned} (\varrho ^2, \textbf{m}^2, \mathcal {E}^2)(t) = (\varrho ^1, \textbf{m}^1, \mathcal {E}^1)(t) \ \text{ for }\ 0 \le t \le T< \infty , \ \mathcal {E}^2(t)< \mathcal {E}^1(t) \ \text{ for }\ T< t < T+\delta . \end{aligned}$$

Next, we compute

5.1 $$\begin{aligned}&\int _0^\infty \exp (-\lambda t) (\mathcal {E}^2 - \mathcal {E}^1)(t) \,\textrm{d} t \nonumber \\&\quad \le \int _T^{T+ \delta } \exp (-\lambda t) (\mathcal {E}^2 - \mathcal {E}^1)(t) \,\textrm{d} t \nonumber \\&\qquad + \int _{T+ \delta }^\infty \exp (-\lambda t) (\mathcal {E}^2 - \mathcal {E}^1)(t) \,\textrm{d} t \nonumber \\&\quad \le \exp (-\lambda (T + \delta )) \left[ \int _T^{T+\delta } (\mathcal {E}^2 - \mathcal {E}^1)(t) \,\textrm{d} t + \frac{2}{\lambda } \mathcal {E}_0 \right] < 0, \end{aligned}$$

provided λ>0 is large enough.

This motivates the following definition.

**Figure:**
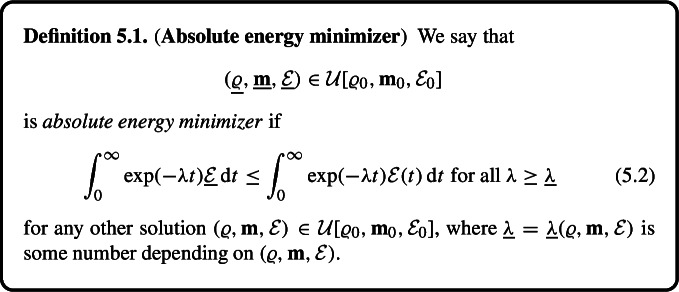

It follows from the previous discussion that any absolute energy minimizer is minimal with respect to the order $\prec _\textrm{loc}$; whence an admissible weak solution of the Euler system.

Next, we report the following variant of Lerch theorem, see [7, Lemma 5.3].

### Lemma 5.2

(**Lerch lemma**) Suppose there exists $\underline{\lambda } > 0$ such that

$$\begin{aligned} \int _0^\infty \exp (-\lambda t) {\mathcal {E}}^1 (t)\,\textrm{d} t = \int _0^\infty \exp (-\lambda t) {\mathcal {E}^2}(t) \,\textrm{d} t \ \text{ for } \text{ all }\ \lambda \ge \underline{\lambda }. \end{aligned}$$

Then

$$\begin{aligned} \mathcal {E}^1 = \mathcal {E}^2. \end{aligned}$$

It follows from Lemma 5.2 that there exists at most one absolute energy minimizer. Indeed it is easy to see that the set of all absolute minimizers is convex. Suppose that are two absolute minimizers $(\underline{\varrho }^1, \underline{\textbf{m}}^1, \underline{\mathcal {E}})$ and $(\underline{\varrho }^2, \underline{\textbf{m}}^2, \underline{\mathcal {E}})$, where

5.3 $$\begin{aligned} \mathcal {E} = \int _{\Omega } E(\varrho ^1, \textbf{m}^1) \ \,\textrm{d} {x} = \int _{\Omega } E(\varrho ^2, \textbf{m}^2) \ \,\textrm{d} {x}. \end{aligned}$$

The convex combination

$$\begin{aligned} (\underline{\varrho }, \underline{\textbf{m}}, \underline{\mathcal {E}}) = \frac{1}{2} (\underline{\varrho }^1, \underline{\textbf{m}}^1, \underline{\mathcal {E}}) + \frac{1}{2} (\underline{\varrho }^2, \underline{\textbf{m}}^2, \underline{\mathcal {E}}) \end{aligned}$$

is another absolute energy minimizers; whence a weak solution satisfying

5.4 $$\begin{aligned} \mathcal {E} = \int _{\Omega } E \left( \frac{1}{2} \underline{\varrho }^1 + \frac{1}{2} \underline{\varrho }^2, \frac{1}{2} \underline{\textbf{m}}^1 + \frac{1}{2} \underline{\textbf{m}}^2 \right) \ \,\textrm{d} {x}. \end{aligned}$$

As *E* is strictly convex, the relations (5.3), (5.4) are compatible only if

$$\begin{aligned} \underline{\varrho }^1 = \underline{\varrho }^2,\quad \underline{{\textbf {m}}}^1 = \underline{{\textbf {m}}}^2. \end{aligned}$$

Let us summarize the previous discussion.

**Figure:**
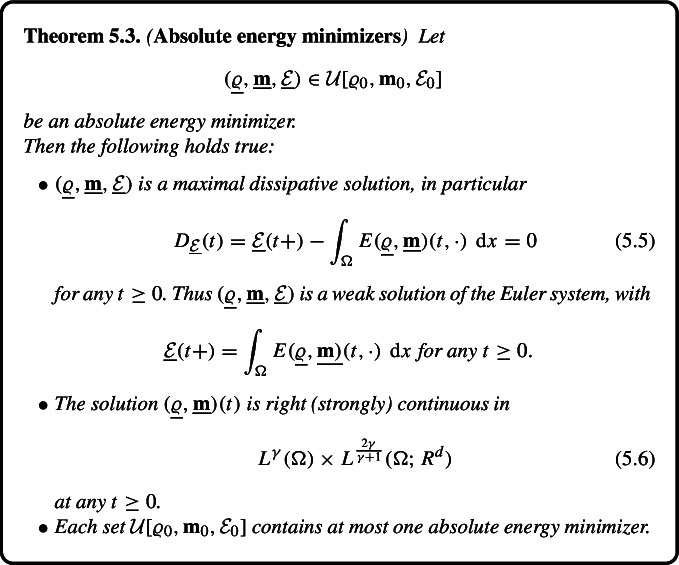

Finally, it follows from inequality (5.1) that any absolute minimizer with respect to order $\prec _\textrm{loc}$ is an absolute energy minimizer. Recall that $(\underline{\varrho }, \underline{\textbf{m}}, \underline{\mathcal {E}}) \in \mathcal {U}[\varrho _0, \textbf{m}_0, \mathcal {E}_0]$ is an absolute minimizer with respect to order $\prec _\textrm{loc}$ if

$$\begin{aligned} (\underline{\varrho }, \underline{\textbf{m}}, \underline{\mathcal {E}}) \prec _\textrm{loc} (\varrho , \textbf{m}, \mathcal {E}) \ \text{ for } \text{ any }\ (\varrho , \textbf{m}, \mathcal {E}) \in \mathcal {U}[\varrho _0, \textbf{m}_0, \mathcal {E}_0], \ (\varrho , \textbf{m}, \mathcal {E}) \ne (\underline{\varrho }, \underline{\textbf{m}}, \underline{\mathcal {E}}). \end{aligned}$$

It is worth noting that the property of being absolute minimizer with respect to $\prec _\textrm{loc}$ is a *local* property that can be defined independently of the actual length of the time interval on which the problem is considered.

Next, we show that the family of absolute energy minimizers, provided they exist for any initial data, enjoy the semigroup properties.

### Proposition 5.4

The following properties hold:

- Suppose
  $$\begin{aligned} (\underline{\varrho }, \underline{\textbf{m}}, \underline{\mathcal {E}}) \in \mathcal {U}[\varrho _0, \textbf{m}_0, \mathcal {E}_0] \end{aligned}$$
  is an absolute energy minimizer. Then its time shift $\mathcal {S}_T (\underline{\varrho }, \underline{\textbf{m}}, \underline{\mathcal {E}})$ is an absolute energy minimizer in $\mathcal {U}[(\underline{\varrho }, \underline{\textbf{m}}, \underline{\mathcal {E}})(T, \cdot )]$.
- Let $(\underline{\varrho }^1, \underline{\textbf{m}}^1, \underline{\mathcal {E}}^1)$ be an absolute energy minimizer in $\mathcal {U}[\varrho _0, \textbf{m}_0, \mathcal {E}_0]$, and let $(\underline{\varrho }^2, \underline{\textbf{m}}^2, \underline{\mathcal {E}}^2)$ be an absolute energy minimizer in $\mathcal {U}[(\underline{\varrho }^1, \underline{\textbf{m}}^1, \underline{\mathcal {E}}^1)(T, \cdot )]$. Then their concatenation
  $$\begin{aligned} (\underline{\varrho }^1, \underline{\textbf{m}}^1, \underline{\mathcal {E}}^1) \cup _T (\underline{\varrho }^2, \underline{\textbf{m}}^2, \underline{\mathcal {E}}^2) \end{aligned}$$
  is an absolute energy minimizer in $\mathcal {U}[\varrho _0, \textbf{m}_0, \mathcal {E}_0]$, meaning
  $$\begin{aligned} (\underline{\varrho }^1, \underline{\textbf{m}}^1, \underline{\mathcal {E}}^1) =(\underline{\varrho }^1, \underline{\textbf{m}}^1, \underline{\mathcal {E}}^1) \cup _T (\underline{\varrho }^2, \underline{\textbf{m}}^2, \underline{\mathcal {E}}^2). \end{aligned}$$

**Fig. 1:**
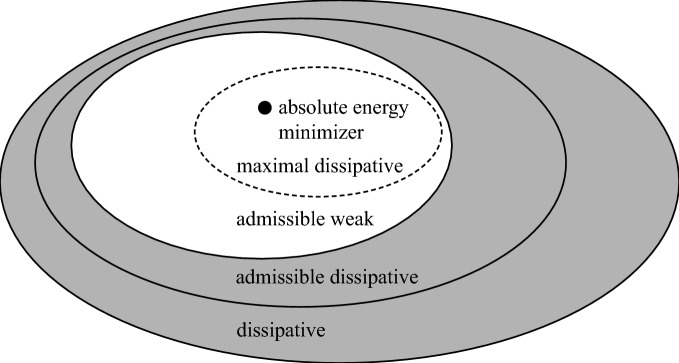
Relations between the various solution concepts. The black dot denotes a solution set with a unique element. The gray color indicates solution sets with at least one element, while the existence of elements of the white sets is open. We prove that any maximal dissipative solution is in fact an admissible weak solution

### Proof

**Shift property.**

Let $(\varrho , \textbf{m}, \mathcal {E}) \in \mathcal {U}[(\underline{\varrho }, \underline{\textbf{m}}, \underline{\mathcal {E}})(T, \cdot )]$ be an arbitrary solution. We have

$$\begin{aligned} \int _0^\infty&\exp (-\lambda t) \mathcal {S}_T \underline{\mathcal {E}}(t) \,\textrm{d} t \equiv \int _0^\infty \exp (-\lambda t) \underline{\mathcal {E}}(t + T) \,\textrm{d} t \\&= \exp (\lambda T) \int _0^\infty \exp (-\lambda (t + T)) \underline{\mathcal {E}}(t + T) \,\textrm{d} t \\  &= \exp (\lambda T) \left[ \int _0^\infty \exp (-\lambda t) \underline{\mathcal {E}}(t) \,\textrm{d} t - \int _0^T \exp (- \lambda t) \underline{\mathcal {E}}(t) \,\textrm{d} t \right] \\&\le \exp (\lambda T) \left[ \int _0^\infty \exp (-\lambda t) [\underline{\mathcal {E}} \cup _T \mathcal {E}](t) \,\textrm{d} t - \int _0^T \exp (- \lambda t) \underline{\mathcal {E}}(t) \,\textrm{d} t \right] \\&= \exp (\lambda T) \int _T^\infty \exp (- \lambda t) \mathcal {E}(t - T) \,\textrm{d} t = \int _0^\infty \exp (- \lambda t) \mathcal {E}(t) \,\textrm{d} t \end{aligned}$$

for a suitable sequence $\lambda \ge \underline{\lambda }$ associated to $(\underline{\varrho }, \underline{\textbf{m}}, \underline{\mathcal {E}}) \cup _T (\varrho , \textbf{m}, \mathcal {E})$.

**Concatenation property.**

A straightforward manipulation yields

$$\begin{aligned} \int _0^\infty&\exp (- \lambda t) [\underline{\mathcal {E}}^1 \cup _T \underline{\mathcal {E}}^2](t) \,\textrm{d} t = \int _0^T \exp (-\lambda t) \underline{\mathcal {E}}^1(t) \,\textrm{d} t + \int _T^\infty \exp (-\lambda t) \underline{\mathcal {E}}^2(t - T) \,\textrm{d} t \\&= \int _0^T \exp (-\lambda t) \underline{\mathcal {E}}^1(t) \,\textrm{d} t + \exp (\lambda T) \int _0^\infty \exp (- \lambda t) \underline{\mathcal {E}}^2 (t) \,\textrm{d} t \\&\le \int _0^T \exp (-\lambda t) \underline{\mathcal {E}}^1(t) \,\textrm{d} t + \exp (\lambda T) \int _0^\infty \exp (- \lambda t) \mathcal {S}_T \underline{\mathcal {E}}^1 (t) \,\textrm{d} t = \int _0^\infty \exp (-\lambda t) \underline{\mathcal {E}}^1(t) \,\textrm{d} t \end{aligned}$$

for all $\lambda \ge \underline{\lambda }$ related to $\mathcal {S}_T \underline{\mathcal {E}}^1$. As a matter of fact, it follows from the previous step and uniqueness of absolute minimizers that

$$\begin{aligned} (\varrho ^2, \textbf{m}^2, \mathcal {E}^2) = \mathcal {S}_T (\varrho ^1, \textbf{m}^1, \mathcal {E}^1). \end{aligned}$$

We conclude that

$$\begin{aligned} \underline{\mathcal {E}}^1 \cup _T \underline{\mathcal {E}}^2 = \underline{\mathcal {E}}^1. \end{aligned}$$

□

## Conclusion

We have identified five different classes of dissipative solutions emanating from given data $(\varrho _0, \textbf{m}_0, \mathcal {E}_0)$ (see Fig. 1):

$$\begin{aligned} \Big \{ \text{ absolute } \text{ energy } \text{ minimizer } \Big \}&\subset \Big \{ \text{ maximal } \text{ dissipative } \text{ solutions } \Big \} \subset \Big \{\text{ admissible } \text{ weak } \text{ solutions }\Big \} \\&\subset \Big \{ \text{ admissible } \text{ dissipative } \text{ solutions } \Big \} \\&\subset \Big \{ \text{ dissipative } \text{ solutions } \Big \}. \end{aligned}$$

Admissible dissipative solutions do exist for any finite energy initial data and admit a semigroup selection resulting from a two step process. Maximal dissipative solutions are admissible weak solution of the Euler system. They comply with Dafermos’ principle of maximal dissipation. Their existence for general initial data, however, remains an open problem. The absolute energy minimizer, provided it exists, is a unique weak solution in $\mathcal {U}[\varrho _0, \textbf{m}_0, \mathcal {E}_0]$.

## Data Availability

Data sharing is not applicable to this article as no data sets were generated during the current study.
